# Gut–Liver Axis: Liver Sinusoidal Endothelial Cells Function as the Hepatic Barrier in Colitis-Induced Liver Injury

**DOI:** 10.3389/fcell.2021.702890

**Published:** 2021-07-16

**Authors:** Yang Wang, Yifan Zhang, Yun Liu, Jun Xu, Yulan Liu

**Affiliations:** ^1^Department of Gastroenterology, Peking University People’s Hospital, Beijing, China; ^2^Clinical Center of Immune-Mediated Digestive Diseases, Peking University People’s Hospital, Beijing, China; ^3^Institute of Clinical Molecular Biology & Central Laboratory, Peking University People’s Hospital, Beijing, China

**Keywords:** gut-liver axis, hepatic barrier, liver sinusoidal endothelial cells, neutrophils, liver injury, colitis, lipopolysaccharide

## Abstract

**Background:**

Based on the gut–liver axis theory, a leaky gut can aggravate liver injury. However, clinical studies suggest that although gut mucosa damage is commonly observed in inflammatory bowel disease (IBD), it seldom leads to severe liver injury. We hypothesize that there is a hepatic barrier in the gut–liver axis, which protects the liver against gut-derived invasive factors.

**Methods:**

Colitis was induced by dextran sulfate sodium (DSS) in eight different liver injury models in Sprague–Dawley rats. Liver sinusoidal endothelial cell (LSEC) injury was evaluated by a scanning and transmission electron microscope. Neutrophils were depleted by injection of anti-rat polymorphonuclear serum. Two pneumonia models were also induced to investigate the mechanism of neutrophil recruitment and activation. LSECs isolated from rat liver were used to investigate the effect on neutrophil recruitment and activation.

**Results:**

Among eight liver injury models, DSS colitis had no effect on liver injury in three models with normal LSECs. In the other five models with LSEC rupture, liver injury was significantly exacerbated by colitis, and increased hepatic neutrophil accumulation was observed. When neutrophils were depleted, colitis-induced liver injury was significantly attenuated. In pneumonia, liver injury, and colitis models, the level of CXCL1 correlated with the recruitment of neutrophils in different tissues, while DSS colitis and LSEC injury synergistically contributed to increased CXCL1 expression in the liver. In colitis-induced liver injury, neutrophils were activated in the liver. Injured LSECs showed both structural and functional changes, with significantly increased expression of CXCL1 and TNF-α under the stimulation of lipopolysaccharide (LPS). The combination of gut-derived LPS and LSEC-derived TNF-α led to the activation of neutrophils, characterized by enhanced production of reactive oxygen species, pro-inflammatory cytokines, and the formation of neutrophil extracellular traps.

**Conclusion:**

LSECs constitute a vitally important barrier in the gut–liver axis, defending the liver against colitis-induced injury. When LSECs are damaged, they can turn into a pro-inflammatory pattern under the stimulation of LPS. LSEC injury and colitis-derived LPS synergistically contribute to the recruitment and activation of hepatic neutrophils. Neutrophils play a pivotal role as a downstream effector in colitis-induced liver injury.

## Introduction

The liver is intimately associated with the intestinal tract. The interactions between the two organs have raised an increasing interest since Marshall proposed the theory of the gut–liver axis (Marshall, 1998). Once the gut barrier is damaged, gut-derived pathogens and bacterial metabolites can translocate to the liver (Ohtani and Kawada, 2019). Under certain conditions, these gut-derived pathogenic factors can induce pathological reactions including liver inflammation, necrosis, and fibrosis, thus contributing to the progression of liver diseases (Seo and Shah, 2012; Wiest et al., 2017).

It is noteworthy that quite a few clinical reports suggest that significantly damaged gut mucosa is commonly observed in active inflammatory bowel disease (IBD) but seldom leads to severe liver injury independently. For instance, in a long-term follow-up study conducted by Gisbert et al. (2007) for IBD patients with a previously healthy liver, only 4.3% (34 out of 786) developed abnormal liver tests during follow-up. Thin et al. (2014) also reported similar liver stiffness values between IBD patients and non-IBD controls, as well as a low incidence of liver disease in IBD patients (6.4%). However, for IBD patients with pre-existing liver diseases, such as non-alcoholic fatty liver disease (NAFLD) and drug-induced liver injury (DILI), these pre-existing liver injuries might be aggravated by the gut barrier damage (Thin et al., 2014). For patients receiving drugs with potential hepatotoxicity, such as methotrexate, the liver adverse events rate was reported to be ranging from 17.5 to 24% in IBD patients (Fournier et al., 2010; Feagan et al., 2014), much higher than that in non-IBD patients (11.2%) (Conway et al., 2015). Animal studies also reveal that dextran sulfate sodium (DSS)-induced colitis does not mediate distinct liver injury independently, despite a leaky gut and elevated portal lipopolysaccharide (LPS) level (Gabele et al., 2011; El Kasmi et al., 2012). In contrast, DSS colitis can exacerbate pre-existing liver diseases [e.g., non-alcoholic steatohepatitis (NASH)] (El Kasmi et al., 2012; Achiwa et al., 2016).

These pieces of evidence raise the hypothesis that there might be an important barrier in the liver, defending against gut-derived pathogenic factors. Since most studies in the field of the gut–liver axis have focused on the gut barrier, the barrier function of the liver remains largely unknown. In consideration of the anatomical location and structure, liver sinusoidal endothelial cells (LSECs) constitute a natural barrier that separates the liver parenchyma from the bloodstream in the sinusoidal lumen (Thomson and Knolle, 2010; Bleau et al., 2016). Thus, in the liver, LSECs are the first in contact with portal-delivered gut-derived pathogenic factors. It has been reported that LSECs help to maintain liver homeostasis *via* mediating immune tolerance (Thomson and Knolle, 2010). LSECs can also remove pathogenic molecules from the circulation through scavenger function (Suzuki et al., 2016; Yao et al., 2016). Furthermore, significant LSEC damage has also been found in several types of liver diseases, such as liver fibrosis and NASH (Miyao et al., 2015; Xing et al., 2016).

So far, it has not been investigated whether LSECs can protect the liver against gut-derived pathogenic factors, especially when the gut barrier is damaged. Besides, the liver is a large organ containing many other types of non-parenchymal cells (Crispe, 2009), including Kupffer cells, B cells, T cells, and natural killer cells (NK). It is unknown which non-parenchymal cell plays the role of barrier and protects the liver against gut-derived invasive factors. Besides, in recent decades, infiltration of neutrophils within the liver has been confirmed in various types of liver diseases including NAFLD and DILI. The effects of neutrophils as well as the hepatic barrier in colitis-induced liver injury need to be further investigated.

In this study, we aimed to explore which type of liver cell constitutes an important aspect of the hepatic barrier in the gut–liver axis and to clarify the role of hepatic barrier and neutrophils in colitis-induced liver injury.

## Materials and Methods

### Animals

Animal experiments were performed using Sprague Dawley rats. Sprague Dawley rats were obtained from Beijing Vital River Laboratory Animal Technology Co., Ltd. Rats were housed in 595 × 380 × 200 mm cages in a temperature-controlled room (21°C ± 2°C) with free access to food and water *ad libitum* unless specified, and maintained on a 12-h dark/light cycle. Animals that died in the process of illness before reaching the time point were excluded. During the experiment, a total of one rat in the DSS + SOS group and two rats in the DSS + SOS + LPSPn group died and were excluded. At the end of the experiment, there were at least six rats at each time point in each experiment group. A randomized drug/vehicle administration strategy was performed using the standard = RAND() function in Microsoft Excel. Coded vials containing the treatment drugs or vehicle were prepared by a third person not involved in the experiment to maintain blinding. All the animals were housed in the same shelf in the same room to minimize potential confounders. All protocols dealing with animals were reviewed and approved by the Ethics Committee of Peking University People’s Hospital (Approval No. 2020PHE074).

### Reagents for Modeling

The following reagents were used in animal modeling: DSS (MPbio), monocrotaline (MCT) (Sigma-Aldrich), CCl_4_ (Aladdin), Concanavalin A (Sigma-Aldrich), 60% high-fat diet (Medicience), methionine and choline deficiency diet (Medicience), LPS (Sigma-Aldrich), HCl (Aladdin), and anti-rat-polymorphonuclear serum (Accurate Chemical & Scientific Corporation).

### Antibodies

The following antibodies were used in this research:

For flow cytometry analysis: anti-rat-CD3 (#201406, BioLegend), anti-rat-CD4 (#201509, BioLegend), anti-rat-CD8a (#201712, BioLegend), anti-rat-CD11b/c (#201817, BioLegend), anti-rat-granulocytes (#550002, BD Pharmingen), and anti-rat-CD68 (MCA341A700, Bio-Rad).

For immunohistochemistry: anti-myeloperoxidase (MPO) rabbit antibody (GB11224, Servicebio, 1:1,000), anti-CD68 rabbit antibody (GB11067, Servicebio, 1:500), anti-CD19 rabbit antibody (GB11061-1, Servicebio, 1:400), anti-CD3 rabbit antibody (GB111337, Servicebio, 1:1,000), anti-rat-endothelial-cell-antibody-1 (RECA-1, ab9774, Abcam, 1:200), and anti-CXCL1 (ab86436, Abcam, 1:200).

For immunocytochemistry: RECA-1 (ab9774, Abcam, 1:400), anti-CXCL1 (ab86436, Abcam, 1:100), and secondary antibodies (goat anti-mouse IgG Alexa Fluor^®^ 488, 1:200; goat anti-rabbit IgG Alexa Fluor 594^®^, 1:200; ZSGB-BIO).

For Western blot: anti-p-p38 (#4511, Cell Signaling Technology, 1:1,000), anti-p38 (#8690, Cell Signaling Technology, 1:1,000), anti-p-p65 (#3033, Cell Signaling Technology, 1:1,000), anti-p65 (#8242, Cell Signaling Technology, 1:1,000), anti-GAPDH (#5174, Cell Signaling Technology, 1:2,000), anti-p47 (PA5-104250, Thermo Fisher Scientific, 1:2,000), and anti-p-p47 (PA5-99359, Thermo Fisher Scientific, 1:1,000).

### Liver Injury Models

As depicted in Supplementary Figure 1, the following eight liver injury models were developed: (1) sinusoidal obstruction syndrome (SOS), induced by oral administration of MCT (160 mg/kg) at day 0; rats were sacrificed on days 1, 3, and 5. (2) DILI, induced by intraperitoneal injection of carbon tetrachloride (CCl_4_, 1 ml/kg) at day 0; rats were sacrificed on day 1, 3, and 5. (3) Liver fibrosis (LF), induced by repeated injection of CCl_4_ from week 0 to week 8 (1 ml/kg, twice per week); rats were sacrificed on weeks 4, 6, and 8. (4) Acute high-dose concanavalin-A (ConA) hepatitis (H-ConA), induced by tail vein injection of ConA at day 0 (37.5 mg/kg); rats were sacrificed on days 1, 3, and 5. (5) Acute low-dose ConA hepatitis (L-ConA), induced by ConA (20 mg/kg) at day 0; rats were sacrificed on days 1, 3, and 5. (6) Chronic ConA hepatitis (C-ConA), induced by weekly repeated injection of ConA (20 mg/kg) from week 0 to week 8; rats were sacrificed on weeks 4, 6, and 8. (7) Non-alcoholic fatty liver (NAFL), fed by 60% high-fat diet; rats were sacrificed on weeks 16, 20, and 24. (8) NASH, fed by methionine and choline-deficient diet for 4 weeks.

### Acute and Chronic DSS Colitis Models

Acute DSS colitis (aDSS) was induced by 5% (w/v) DSS dissolved in the autoclaved drinking water for 7 days. Animals were sacrificed at the end of DSS consumption. For acute DSS colitis + acute liver injury groups (including SOS, DILI, H-ConA, and L-ConA), DSS was started 7 days prior to liver injury modeling as shown in Supplementary Figure 1. Chronic DSS colitis (cDSS) was induced by multiple DSS cycles (each cycle included 6 days of 5% DSS followed by 8 days of water) and rats were sacrificed in week 8. For chronic DSS colitis + chronic liver injury groups (including LF and C-ConA), DSS/water cycle was started 4 weeks before liver injury modeling (Supplementary Figure 1) and administered throughout the course of liver injury modeling. For the chronic DSS colitis + NAFL group, since the malnutrition due to diarrhea could significantly prevent the modeling of fatty liver, the DSS/water cycle was given since week 12 when fatty liver was successfully generated. For the chronic DSS colitis + NASH group, colitis and liver injury were simultaneously induced for only 4 weeks, since DSS + NASH modeling more than 4 weeks could lead to the significantly elevated death rate of rats. Rats in matched control (NC group) consumed water drinking only.

### Neutrophil Depletion

To investigate the role of neutrophils in colitis-aggravated liver injury, neutrophil depletion was further performed by tail vein injection of anti-rat-polymorphonuclear serum (1 ml/kg) 1 day before liver injury modeling in SOS and DILI models, as depicted in Supplementary Figure 2. Animals were sacrificed and hepatic pathology was evaluated on day 3 and day 1 for SOS and DILI, respectively.

### Pneumonia Models

As shown in Supplementary Figure 3, to explore how neutrophils were recruited into the liver and other organs, we also introduced LPS-induced pneumonia (LPSPn, which had a significantly increased number of peripheral blood neutrophils) *via* intratracheal administration of LPS (5 mg/kg) at day 1. We also induced another pneumonia model, hydrochloric acid-induced pneumonia (HClPn) *via* intratracheal administration of hydrochloric acid (0.1 N, pH = 1, dose: 1 ml/kg) at day 1. HClPn differs from LPSPn since it is a simple type of chemical pneumonitis without the effect of LPS. LPSPn and HClPn models were combined with SOS and DSS colitis models to investigate the mechanism of neutrophil recruitment. Animals were sacrificed on day 3, and the number of neutrophils in the peripheral blood, liver, lung, and colon was examined.

### LSEC Isolation and Culture

Primary LSECs were isolated from the rat liver according to the protocol previously described (Braet et al., 1994). Briefly, rats were anesthetized, and perfusion with HBSS followed by collagenase IV (Sigma-Aldrich) solution (0.5 mg/ml) *via* the portal vein was performed. After further disrupting and digesting, the cell suspension was then filtered through nylon gauze (mesh 100). Non-parenchymal cells enriched with LSECs were obtained by Percoll density gradient centrifugation and resuspended in a culture medium. Primary LSECs were further purified by selective adherence of Kupffer cells on dishes without coating. Isolated LSECs were cultured in RPMI-1640 (Thermo Fisher Scientific) with 100 U/ml penicillin, 100 μg/ml streptomycin, and 0.5% FBS. LSECs were seeded in 24-multiwell dishes or glass coverslips coated by rat tail tendon collagen (Solarbio) and cultured at 37°C with 5% CO_2_. In the experiment, LSECs were incubated with 4 mM MCT for 4 h to induce LSEC injury or vehicle (PBS) for control. LPS (Sigma-Aldrich, 100 ng/ml) or vehicle (PBS) was also used for stimulating LSECs. After 4 h, cell culture supernatant was collected, and the culture medium was changed. LSECs were then incubated for another 12 h. At 16 h, cell culture supernatant was collected and frozen; cell monolayers were collected for further RNA and protein extraction.

### Neutrophil Isolation and Culture

In order to explore where neutrophils were activated, we isolated neutrophils in the peripheral blood and portal vein by using a blood neutrophil separation kit (Solarbio) according to the manufacturer’s instructions. Hepatic neutrophils were isolated *via* fluorescence-activated cell sorting (sorting strategy shown in Supplementary Figure 4). The morphology of isolated neutrophils was evaluated by using Giemsa staining. The purity of neutrophils was tested by flow cytometry (CD11b/c^+^SSA^high^) and immunocytochemistry for MPO. To study the mechanism of neutrophil activation, co-culture experiments were performed as shown in Supplementary Figure 5. Neutrophils were isolated from the peripheral blood of rats with different diseases and then incubated with stimuli including LPS (Sigma-Aldrich, 100 ng/ml), TNF-α (#CR38, Novoprotein, 100 ng/ml), or with the supernatants of LSECs treated by different reagents (vehicle, LPS, MCT, and MCT + LPS). Activation of neutrophils was estimated by flow cytometry of reactive oxygen species (ROS), Western blot examination of p38 phosphorylation, and expression of pro-inflammatory cytokines. Moreover, the formation of neutrophil extracellular traps (NETs) was also tested by immunofluorescence of extracellular DNA and scanning electron microscopy (SEM). Rat intestinal content containing bacteria was also collected and added into the medium, and the bacteria-trapping effect of NETs was detected by SEM. For the Transwell experiment of neutrophils, recombinant rat CXCL1 (PR3004, EBT SYSTEMS, ranging from 100 to 1,000 pg/ml) was used as the neutrophil attractant chemokine for positive control.

### Statistical Analysis

Data are presented as means ± standard error. Statistical analysis was performed with SPSS 17.0 for Windows. Statistical difference between groups was determined by the Student’s *t*-test or analysis of variance when appropriate.

## Results

### DSS Colitis Can Lead to Increased Liver Injury When LSECs are Damaged

**FIGURE 1:**
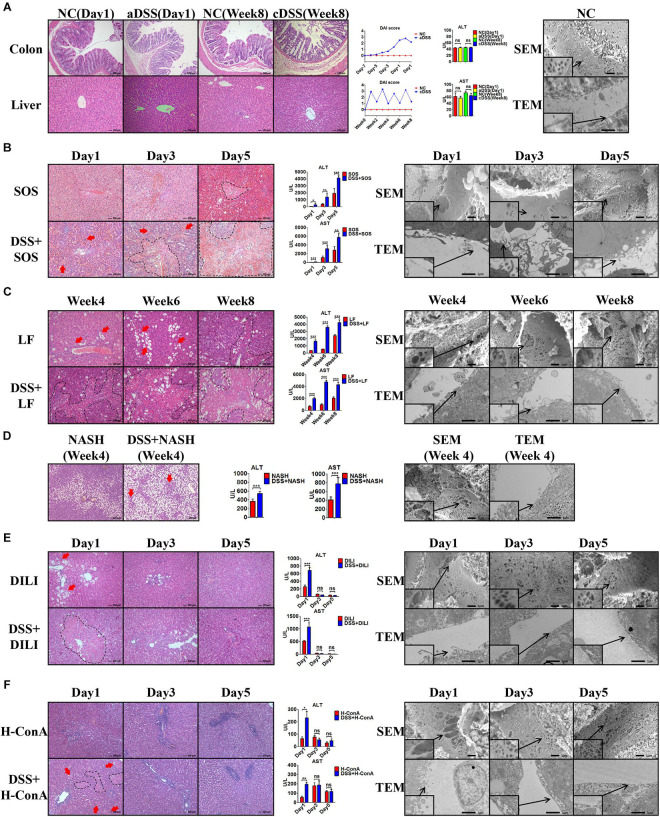
Colitis aggravates liver injury when LSECs are damaged. **(A)** H&E staining was used to investigate the pathological findings of colon and liver in normal control (NC), acute dextran sulfate sodium (DSS) colitis (aDSS) group, and chronic DSS colitis (cDSS) group. Disease Activity Index (DAI) score was used to evaluate the severity of colitis during the course of disease. Alanine aminotransferase (ALT) and aspartate aminotransferase (AST) were tested to evaluate liver injury. Scanning electron microscopy (SEM) and transmission electron microscopy (TEM) showed the ultrastructural features of normal liver sinusoidal endothelial cells (LSECs). The effect of DSS colitis on liver injury and the damage of LSECs were also evaluated in **(B)** the sinusoidal obstruction syndrome (SOS) model; **(C)** the liver fibrosis (LF) model; **(D)** the non-alcoholic steatohepatitis (NASH) model; **(E)** the drug-induced liver injury (DILI) model; and **(F)** the high-dose ConA hepatitis (H-ConA) model. ns, non-significant; *p < 0.05; **p < 0.01; ***p < 0.001 by *t*-test; *n* = 6 at each time point per group; figures are representative of three experiments.

In the healthy liver (Figure 1A) with normal LSECs, neither acute DSS colitis (aDSS) nor chronic DSS colitis (cDSS) led to liver injury. Although there were significant intestinal mucosa damage, elevated disease activity index score (Figure 1A), and increased LPS level in the portal vein blood (Supplementary Figure 6) in DSS colitis, the liver histology on H&E staining and liver enzymes were normal (Figure 1A). Normal LSECs on SEM and TEM imaging were characterized by the fenestrae well-organized as sieve plates on the sinusoidal endothelium and intact LSECs lining between the space of Disse and the sinusoidal lumen (Figure 1A).

Among the eight liver injury models, there was sustained LSEC injury in three models, namely, SOS (Figure 1B), LF (Figure 1C), and NASH (Figure 1D). Distinct LSEC injury was observed throughout the course of the disease, as characterized by the remarkable formation of large gaps on liver sinusoidal endothelium on SEM images and the detachment or deficiency of LSECs lining on TEM images. The addition of DSS colitis in these three models led to significantly increased liver necrosis area on H&E staining (red arrows and black dotted lines) as well as elevated ALT and AST levels. Thus, DSS colitis can induce more severe liver injury when LSECs are damaged.

Intriguingly, we observed temporary LSEC injury in DILI (Figure 1E) and acute high-dose ConA hepatitis (H-ConA, Figure 1F). In both models, there were large gaps on sinusoidal endothelium and LSECs lining detachment at day 1, indicating significant LSEC rupture. It turned out that DSS colitis also led to enlarged hepatic necrosis area as well as increased liver enzyme levels at day 1. However, at day 3 and day 5 in both models, SEM and TEM showed well-organized fenestrae on LSECs and intact LSECs lining, suggesting that LSEC injury was recovered. Instead, DSS colitis could no longer aggravate liver injury in both models during days 3 and 5.

In another three liver injury models, namely, L-ConA (Supplementary Figure 7A), C-ConA (Supplementary Figure 7B), and NAFL (Supplementary Figure 7C), the features of LSECs were almost the same with healthy LSECs, indicating that there was no significant LSEC injury in all three models. DSS colitis had no effect on the severity of liver injury since there were no significant differences concerning the liver necrotic lesion on H&E staining and liver enzyme level. These results prove that DSS colitis can aggravate liver injury when LSECs are damaged.

### Significant Accumulation and Infiltration of Hepatic Neutrophils in Colitis-Induced Liver Injury

**FIGURE 2:**
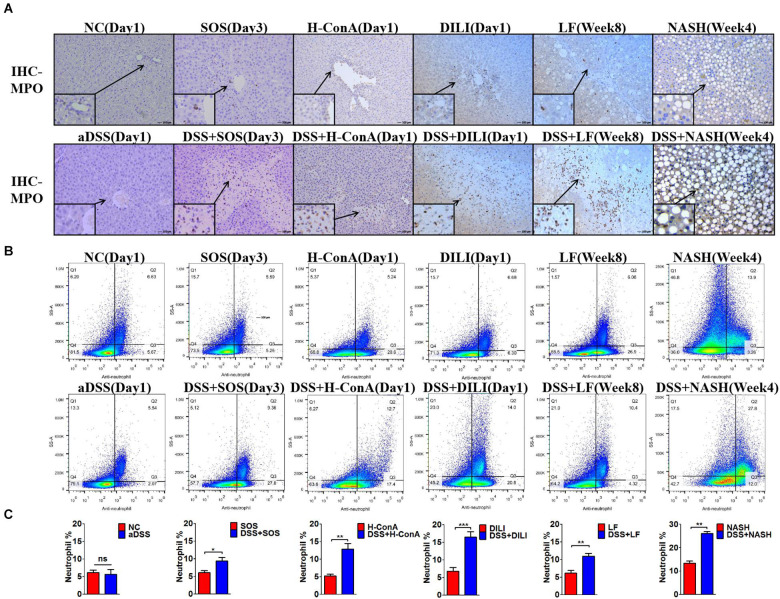
Increased hepatic neutrophils were observed in colitis-induced liver injury. The infiltration and number of hepatic neutrophils were assessed by **(A)** immunohistochemistry (IHC) for myeloperoxidase (MPO) and **(B,C)** flow cytometry (anti-neutrophil^+^SSA^high^) in the following groups: normal control (NC), acute dextran sulfate sodium (DSS) colitis (aDSS), sinusoidal obstruction syndrome (SOS), DSS + SOS, high-dose ConA hepatitis (H-ConA), DSS + H-ConA, drug-induced liver injury (DILI), DSS + DILI, liver fibrosis (LF), DSS + LF, non-alcoholic steatohepatitis (NASH), and DSS + NASH. ns, non-significant; *p < 0.05; **p < 0.01; ***p < 0.001 by *t*-test; *n* = 5–6 in each group; figures are representative of three experiments.

We further evaluated the changes of hepatic immunocytes in the aforementioned liver injury models that could be aggravated by DSS colitis. Immunohistochemistry (IHC) of MPO revealed that DSS colitis significantly enhanced the hepatic neutrophil infiltration in these models (Figure 2A). The neutrophils were mainly infiltrated within the necrosis zones. In line with the findings above, flow cytometry also revealed significantly increased percentages of hepatic neutrophils (anti-neutrophil^+^SSA^high^) in DSS + SOS (day 3), DSS + H-ConA (day 1), DSS + DILI (day 1), DSS + LF (week 8), and DSS + NASH (week 4) groups, as compared with SOS (day 3), H-ConA (day 1), DILI (day 1), LF (week 8), and NASH (week 4) groups, respectively (*p* < 0.05 for all, Figures 2B,C). The fold changes of neutrophils ranged from 1.5 to 2.46. We also investigated the changes of T cells, B cells, and macrophages. IHC of CD3 and CD19 showed that there was no increased infiltration of T cells and B cells in colitis-aggravated liver injury groups (Supplementary Figures 8A,B). The infiltration of macrophages was evaluated by IHC of CD68. In the SOS model (day 3), there was slightly increased macrophage infiltration within the necrotic lesion in the DSS + SOS group (Supplementary Figure 8C), yet not as significant as neutrophil infiltration. In the other models including H-ConA (Day 1), DILI (Day 1), and LF (week 8), DSS colitis had no effect on the infiltration of macrophages. On the other hand, the neutrophil infiltration in DSS + DILI and DSS + H-ConA groups was most severe at day 1 (Figure 2A) and was gradually attenuated from day 3 to day 5 (Supplementary Figures 8D,E). Flow cytometry also showed similar percentages of CD4^+^ T cells, CD8^+^ T cells, and macrophages between liver injury groups and DSS + liver injury groups (Supplementary Figure 9). In brief, in colitis-aggravated liver injury, neutrophils are the major immunocytes that significantly accumulate and infiltrate the liver.

### Colitis-Induced Liver Injury Is Attenuated When Neutrophils are Depleted

Given that increased hepatic neutrophil accumulation was observed in colitis-induced liver injury, we further verified the role of neutrophils in the SOS model and DILI models. After neutrophils were depleted (Ndep) by anti-rat-neutrophil serum, hepatic neutrophil accumulation and infiltration in Ndep-DSS + SOS and Ndep-DSS + DILI groups were abolished, as demonstrated by IHC-MPO and flow cytometry (Supplementary Figures 10A–D).

**FIGURE 3:**
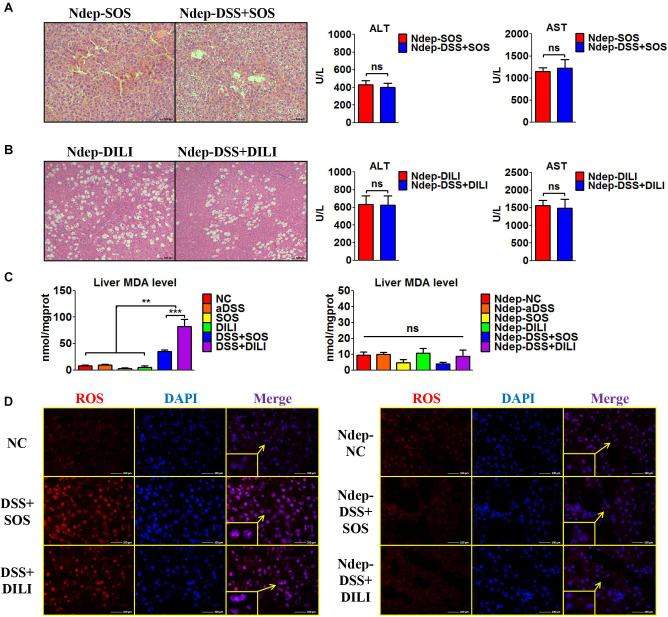
Neutrophil depletion abrogates colitis-induced liver injury. In the sinusoidal obstruction syndrome (SOS) model and the drug-induced liver injury (DILI) model, neutrophils were depleted (Ndep) and experiments were repeated. **(A)** H&E staining of Ndep-SOS model and liver enzymes [including alanine aminotransferase (ALT) and aspartate aminotransferase (AST)] showed that dextran sulfate sodium (DSS) colitis could no longer aggravate liver injury. **(B)** Similar results were found in the DILI model. **(C)** The malondialdehyde (MDA) level in the liver showed a similar trend change with the number of hepatic neutrophils. **(D)** Immunofluorescence staining of reactive oxygen species (ROS) (red) also showed good correlation with the number of hepatic neutrophils. ns, non-significant; **p < 0.01; ***p < 0.001 by one-way ANOVA; *n* = 6 in each group; panels A to C are representative of three experiments; panel D is representative of two experiments.

Interestingly, DSS colitis no longer led to deteriorated liver injury in neutrophil-depleted rats. H&E staining showed similar pathological features between the Ndep-SOS group and the Ndep-DSS + SOS group, as characterized by the hemorrhage in the liver sinusoids and damage of vascular endothelium (Figure 3A). There was no significant difference between the Ndep-SOS group and the Ndep-DSS + SOS group at day 3 in terms of ALT (429 vs. 402 U/L, *p* = 0.69) and AST (1,145 vs. 1,228 U/L, *p* = 0.701) (Figure 3A). Consistently, DSS colitis did not lead to increased liver injury in the Ndep-DSS + DILI group (Figure 3B). Besides, MDA levels were increased in the DSS + SOS group and the DSS + DILI group (Figure 3C). However, without neutrophils, MDA was reduced to normal levels in the Ndep-DSS + SOS group and the Ndep-DSS + DILI group (Figure 3C). The total ROS level in the liver (Figure 3D and Supplementary Figures 10E,F) was found to be consistent with the number of neutrophils and the level of hepatic MDA, indicating that neutrophils also contributed to oxidative stress injury of hepatocytes. These results suggest that in colitis-induced liver injury, neutrophil might be a downstream executor that induces the lethal effect on hepatocytes.

### DSS Colitis and LSEC Injury Synergistically Contribute to Hepatic CXCL1 Expression and Neutrophil Recruitment

**FIGURE 4:**
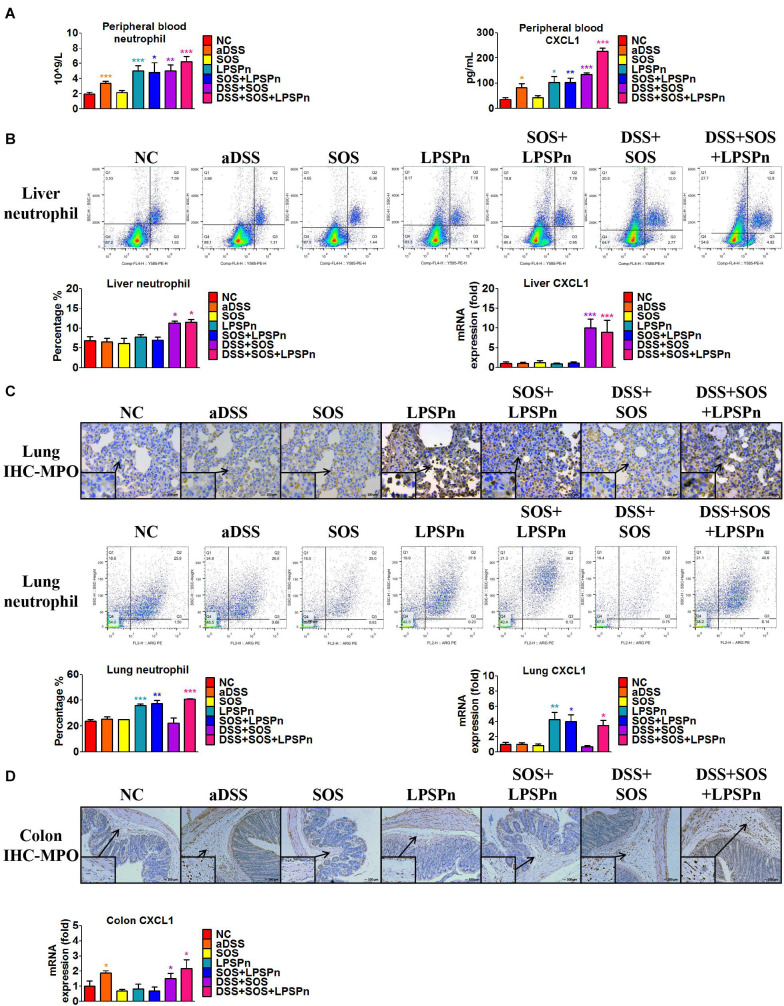
Infiltration and accumulation of neutrophils are related with the expression of CXCL1 in various tissues. The number of neutrophils and expression of CXCL1 were evaluated at day 3 in the following seven groups: normal control (NC), acute dextran sulfate sodium colitis (aDSS), sinusoidal obstruction syndrome (SOS), lipopolysaccharide-induced pneumonia (LPSPn), SOS + LPSPn, DSS + SOS, and DSS + SOS + LPSPn. **(A)** In the peripheral blood, a whole blood cell test was performed and CXCL1 was tested by enzyme-linked immunosorbent assay. **(B)** In the liver, the number of neutrophils was evaluated by flow cytometry (anti-neutrophil^+^SSA^high^), and mRNA level of CXCL1 was tested. **(C)** In the lung, immunohistochemistry for myeloperoxidase (MPO) and flow cytometry were performed and mRNA level of CXCL1 was tested. **(D)** In the colon, immunohistochemistry for MPO and mRNA level of CXCL1 were tested. Asterisk indicates that the level is significantly different from normal control. *p < 0.05; **p < 0.01; ***p < 0.001 by *t*-test; *n* = 5–6 per group; figures are representative of three experiments.

We also discovered an elevated number of peripheral blood neutrophils (Figure 4A) in the DSS + SOS group, which indicated that the increased hepatic neutrophils might be recruited from the peripheral blood. However, increased peripheral neutrophils can be observed in many clinical diseases (e.g., pneumonia), but rarely leads to significant hepatic infiltration and liver injury. To investigate the mechanism of neutrophil recruitment, we induced pneumonia *via* intratracheal administration of LPS (LPSPn) (Supplementary Figure 11), which also had an increased number of peripheral blood neutrophils (Figure 4A). The number of neutrophils and level of CXCL1 in the peripheral blood (Figure 4A), liver (Figure 4B), lung (Figure 4C), and colon (Figure 4D) were evaluated in the following seven groups: NC, aDSS, SOS, LPSPn, SOS + LPSPn, DSS + SOS, and DSS + SOS + LPSPn.

In the liver, lung, colon, and peripheral blood, the number of neutrophils had a good correlation with the level of CXCL1. For instance, in the lung, only three groups with LPS-induced pneumonia (LPSPn, SOS + LPSPn, and DSS + SOS + LPSPn) had elevated pulmonary CXCL1 expression and number of neutrophils, while the other groups did not (Figure 4C). As for the colon, only three groups with DSS-induced colitis (aDSS, DSS + SOS, and DSS + SOS + LPSPn) showed increased CXCL1 and neutrophil infiltration in the colon (Figure 4D). Furthermore, there were increased levels of CXCL1 and the number of neutrophils in the peripheral blood (Figure 4A) in the groups containing LPS-induced pneumonia or DSS-induced colitis (including aDSS, LPSPn, SOS + LPSPn, DSS + SOS, and DSS + SOS + LPSPn). These results suggest that neutrophil recruitment might be mainly due to increased expression of CXCL1.

Notably, neutrophils in the peripheral blood in the aDSS group and the LPSPn group were elevated (Figure 4A), but the neutrophils in the liver remained at normal levels (Figure 4B). This indicated that although neutrophils in the peripheral blood could be increased in various diseases, they did not always enter the liver. However, as compared with the SOS group, the DSS + SOS group had significantly increased hepatic neutrophils and liver CXCL1 expression (Figure 4B), which indicated that when the LSEC barrier was damaged, DSS colitis might lead to the recruitment of hepatic neutrophils *via* CXCL1. In contrast, the SOS + LPSPn group also had elevated peripheral blood neutrophils (Figure 4A), but the hepatic neutrophils and liver CXCL1 expression were within a normal level (Figure 4B). Moreover, when DSS colitis was added (DSS + SOS + LPSPn group), hepatic neutrophils and CXCL1 were thus significantly increased. This further demonstrated that DSS colitis and LSEC barrier damage could synergistically contribute to hepatic CXCL1 expression, which further promoted the recruitment of neutrophils into the liver.

In brief, pneumonia, liver injury, and colitis models demonstrate that the chemokine CXCL1 is strongly correlated with the recruitment of neutrophils. In the liver, CXCL1 expression and neutrophil recruitment are mainly triggered by the synergistic effect of DSS colitis + LSEC injury.

### In Colitis-Induced Liver Injury, Neutrophils are Activated in the Liver

**FIGURE 5:**
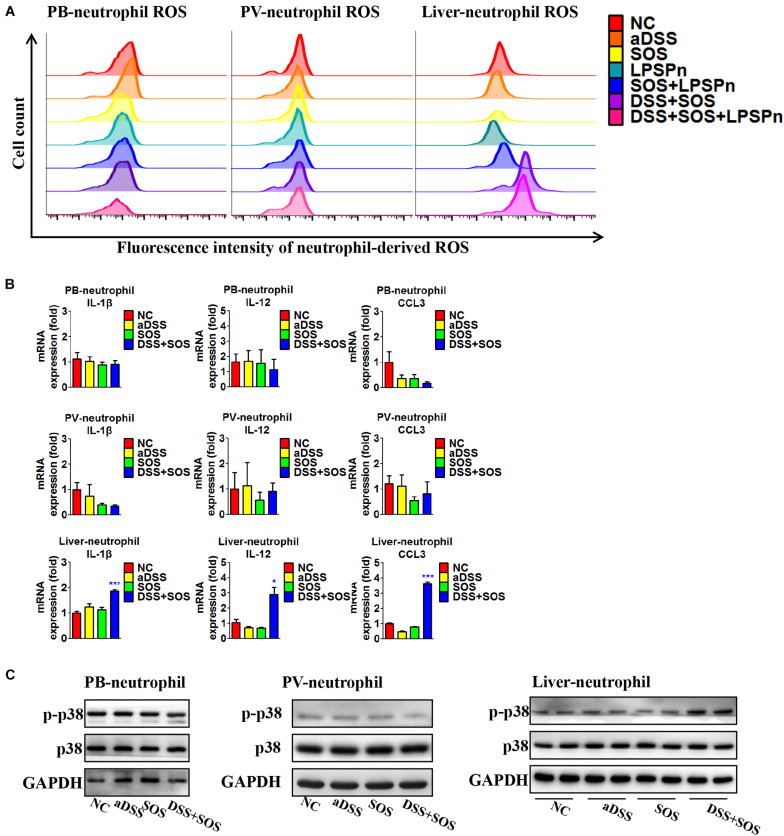
In colitis-induced liver injury, neutrophils are activated in the liver. The activation status of neutrophils was evaluated *via* neutrophil-derived reactive oxygen species (ROS), neutrophil-derived cytokines, and phosphorylation of p38. **(A)** Neutrophil-derived ROS in the peripheral blood (PB), portal vein (PV), and liver were tested *via* flow cytometry in the following seven groups: normal control (NC), acute dextran sulfate sodium colitis (aDSS), sinusoidal obstruction syndrome (SOS), lipopolysaccharide-induced pneumonia (LPSPn), SOS + LPSPn, DSS + SOS, and DSS + SOS + LPSPn. **(B)** Neutrophil-derived cytokines in the peripheral blood, portal vein, and the liver were evaluated in NC, aDSS, SOS, and DSS + SOS groups. **(C)** The phosphorylation of neutrophil p38 was also tested in the peripheral blood, portal vein, and liver. Asterisk indicates that the level is significantly different with normal control. *p < 0.05; ***p < 0.001 by *t*-test; *n* = 5–6 per group; panels A and B are representative of three experiments; panel C is representative of two experiments.

Neutrophils need to be activated so as to induce lethal effects. Thus, we also investigated the activation status of neutrophils in the liver, peripheral blood, and portal vein, respectively. The purity of neutrophils isolated from the liver and blood was confirmed by Giemsa staining (Supplementary Figures 12A,B), flow cytometry (Supplementary Figure 12C), and immunocytochemistry (Supplementary Figures 12D,E). It turned out that the neutrophils in the peripheral blood and portal vein were both inactive, since the production of ROS (Figure 5A), pro-inflammatory cytokines (Figure 5B), and phosphorylation of p38 MAPK (Figure 5C) remained unchanged, while hepatic neutrophils showed elevated production of ROS (Figure 5A, right shift of the purple and pink wave peeks) in colitis-induced liver injury (DSS + SOS and DSS + SOS + LPSPn groups), as well as the upregulation of pro-inflammatory cytokines including IL-1β, IL-12, and CCL3 (Figure 5B), and phosphorylation of p38 MAPK (Figure 5C). Taken together, in colitis-induced liver injury, neutrophils are mainly activated in the liver.

### With the Stimulation of LPS, Damaged LSECs Express Elevated CXCL1 and TNF-α

The above results indicated that both colitis-derived factor and LSEC damage had critical effects on the recruitment and activation of hepatic neutrophils. Firstly, we found that the concentration of LPS in the portal vein was significantly increased in the DSS colitis group as compared to the normal control and LPS-induced pneumonia groups (Supplementary Figure 6). Thus, DSS colitis could lead to a higher concentration of LPS in the portal vein, which would finally enter the liver sinusoid and encounter LSECs.

**FIGURE 6:**
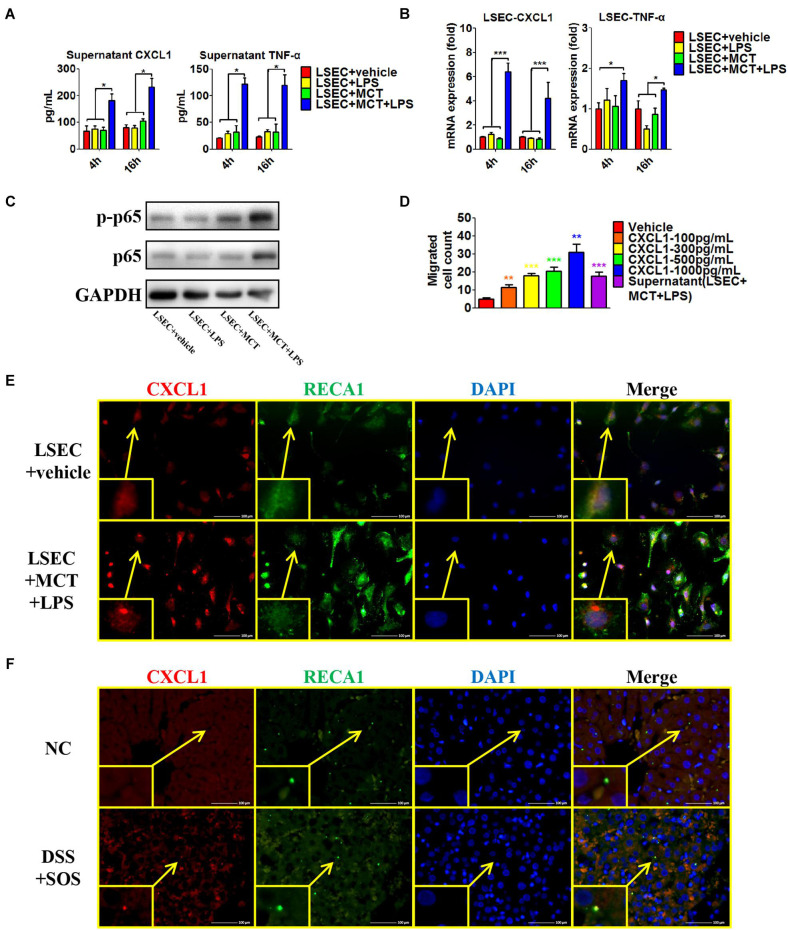
Pro-inflammatory phenotype of damaged LSECs. After LSECs were treated with different stimuli [vehicle, lipopolysaccharide (LPS), monocrotaline (MCT), and MCT + LPS], the following parameters were examined: **(A)** the supernatant CXCL1 and TNF-α, tested by ELISA; **(B)** mRNA levels of CXCL1 and TNF-α; **(C)** phosphorylation of LSEC p65. **(D)** The influence of CXCL1 on neutrophil chemotaxis was evaluated by the number of trans-membrane migrated neutrophils in the Transwell experiment. **(E)** LSEC-derived CXCL1 was also examined by immunocytochemistry of CXCL1 (red) and RECA-1 (green, a marker of rat endothelial cells). **(F)** The co-localization of CXCL1 (red) and RECA-1 (green) was detected by immunohistochemistry in colitis-induced liver injury. Asterisk indicates that the level is significantly different from normal control. *p < 0.05; **p < 0.01; ***p < 0.001 by one-way ANOVA; *n* = 6 in each group; panels **(A–C)** are representative of three experiments; panel **(C)** is representative of one experiment, while other panels are representative of three experiments.

Secondly, we isolated LSECs from normal control rats, and MCT was used to induce the damage of LSECs *ex vivo*, as demonstrated by the remarkable loss of fenestrae and formation of large gaps on SEM imaging (Supplementary Figure 13A). We treated LSECs with (1) vehicle, (2) LPS (100 ng/ml), (3) MCT (4 mM), and (4) MCT (4 mM) + LPS (100 ng/ml). Of the six neutrophil-chemoattractant chemokines, expression and secretion of CXCL1 were significantly increased in the LSEC + MCT + LPS group (Figures 6A,B and Supplementary Figures 13B,C). Besides, cytokines, such as IL-33, TNF-α, and IFN-γ have been reported to be able to activate neutrophils (Zou et al., 2011; Yazdani et al., 2017). We found that only the expression of TNF-α was elevated in the LSEC + MCT + LPS group (Figures 6A,B). With the stimulation of LPS, injured LSECs showed enhanced phosphorylation of p65 (Figure 6C), an important transcription factor involved in many inflammatory processes. We also observed the ability of CXCL1 as well as the supernatant of LSEC (in the LSEC + MCT + LPS group) to promote neutrophil chemotaxis (Figure 6D). Immunofluorescence co-localization of CXCL1 and RECA1 (rat endothelial cell antibody which could bind to LSECs membrane antigen) also identified the positive expression of CXCL1 in the LSEC + MCT + LPS group (Figure 6E and Supplementary Figure 13D). Thus, when LSECs are damaged, the stimulation by LPS can lead to distinct changed function phenotype, characterized by elevated expression of pro-inflammatory mediators including CXCL1 and TNF-α.

Furthermore, we explored the co-localization of CXCL1 and LSECs in the rat liver (Figure 6F and Supplementary Figure 13E). CXCL1 was almost absent in the liver in the NC, aDSS, and SOS groups, while there was positive staining of CXCL1 in the DSS + SOS group. Notably, the location of CXCL1 was spatially associated with LSECs, suggesting that hepatic CXCL1 in the DSS + SOS group might be mainly produced by LSECs. Thereby, LSEC-derived CXCL1 contributed to the recruitment of hepatic neutrophils in the DSS + SOS group.

### LSEC Injury and LPS Synergistically Contribute to Neutrophil Activation

**FIGURE 7:**
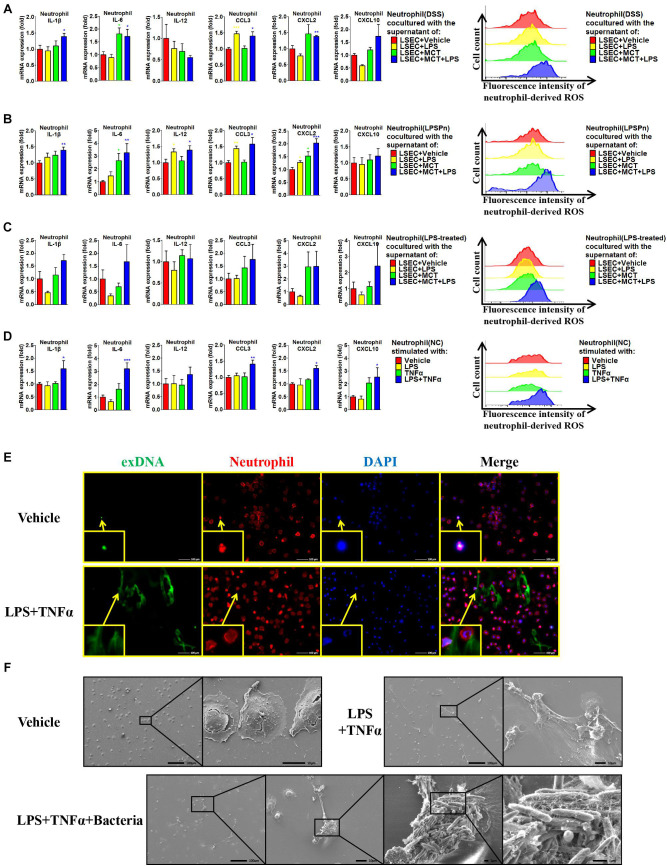
Liver sinusoidal endothelial cells (LSECs) and lipopolysaccharide (LPS) contribute to neutrophil activation. The activation status of neutrophils was evaluated *via* neutrophil-derived cytokines and neutrophil-derived reactive oxygen species (ROS) in the following experiments: **(A)** neutrophils were isolated from dextran sulfate sodium (DSS) colitis rats and incubated with the supernatants of LSECs under different treatments [vehicle, LPS, monocrotaline (MCT), and MCT + LPS]; **(B)** neutrophils were isolated from LPS-induced pneumonia (LPSPn) rats and incubated with the supernatants of LSECs; **(C)** neutrophils from normal control (NC) rats were pre-treated with LPS and then incubated with the supernatants of LSECs; **(D)** neutrophils isolated from NC rats were stimulated with LPS, TNF-α, or the combination treatment. **(E)** Neutrophil extracellular traps (NETs) were detected by immunocytochemistry of neutrophil-derived extracellular DNA (exDNA, green). **(F)** Scanning electron microscopy was used to evaluate the morphology of NETs and its function of capturing bacteria. Asterisk indicates that the level is significantly different with normal control. *p < 0.05; **p < 0.01; ***p < 0.001 by *t*-test; *n* = 6 in each group; figures are representative of three experiments.

Given that hepatic neutrophils were only activated in the DSS + SOS group, we further investigated the effect of LSEC injury and LPS on neutrophil activation. Firstly, we performed co-culture experiments by using peripheral neutrophils from different disease models (NC, DSS colitis, and LPSPn) and the supernatants from LSECs undergoing different treatments as described above (LSEC + vehicle, LSEC + LPS, LSEC + MCT, and LSEC + MCT + LPS). It should be noted that there was a medium change when LSECs were cultured with/without LPS for 4 h (Supplementary Figure 5) so that there should be no confounding molecules, such as LPS in the supernatants. It turned out that neutrophils isolated from the NC group could not be activated by the supernatants from LSECs, since there were no elevated pro-inflammatory cytokines and ROS production in neutrophils (Supplementary Figure 14A). However, neutrophils isolated from the DSS group and the LPSPn group could be activated by the supernatant from the LSEC + MCT + LPS group, as demonstrated by enhanced production of pro-inflammatory cytokines and ROS (Figures 7A,B).

Since the DSS model and LPSPn model are both associated with the effect of LPS, it is unknown whether the effect of LPS or the other disease-related factors contributes to the activation of neutrophils. Therefore, we then induced another pneumonia model, hydrochloric acid-induced pneumonia (HClPn), which was not directly associated with the effect of LPS. We found that neutrophils from HClPn rats showed non-responsiveness to the supernatants from LSECs (Supplementary Figure 14B). It seemed that LPS together with LSECs played an important role in the activation of neutrophils. To further demonstrate the effect of LPS, NC neutrophils were pre-treated with LPS (100 ng/ml) for 2 h; afterward, these neutrophils could be activated by the supernatant from the LSEC + MCT + LPS group (Figure 7C). Lastly, we isolated neutrophils from NC rats and found that normal neutrophils showed non-responsiveness to the single stimulation by LPS or TNF-α. However, the combination of LPS + TNF-α could lead to significantly increased pro-inflammatory cytokines and ROS of neutrophils (Figure 7D).

Besides, the activated neutrophils in the above groups also showed elevated phosphorylation of p38 (Supplementary Figure 14C). Neutrophils treated with LPS + TNF-α also showed enhanced expression and phosphorylation of p47, indicating the synthesis and activation of the neutrophil NADPH oxidase. Remarkable formation of NETs was observed in neutrophils activated by LPS + TNF-α, as demonstrated by immunocytochemistry (Figure 7E and Supplementary Figure 14D) and SEM (Figure 7F and Supplementary Figure 14E). SEM also confirmed the ability of NETs to capture bacteria (Figure 7F). In brief, these results suggest that LPS and TNF-α are two key factors that activate neutrophils.

### Damaged LSECs and Gut-Derived LPS Synergistically Contribute to Hepatic Neutrophil Recruitment and Activation and Promote Liver Injury

A leaky gut can lead to the translocation of not only LPS but also other metabolites and bacterial products. It remains unclear if LPS alone is responsible for colitis-induced liver injury. Thus, we also induced the PVLPS model by portal infusion of LPS. We found that the dose of LPS larger than 100 ng/kg led to the unwanted confounding effect of liver injury (Supplementary Figure 15A), considering DSS colitis did not mediate liver injury independently. Therefore, we selected the dose of 100 ng/kg to study the role of gut-derived LPS. Although portal infusion of LPS (100 ng/kg) did not lead to liver injury, it significantly enhanced the severity of liver injury in combination with the SOS model (Supplementary Figures 15B,C). Damaged LSECs and portal infusion of LPS synergistically promoted the mRNA level of liver CXCL1 (Supplementary Figure 15D), thereby contributing to the recruitment of hepatic neutrophils (Supplementary Figures 15E,F). The hepatic neutrophils in the PVLPS + SOS group also showed elevated production of ROS (Supplementary Figure 15G), indicating the activation status of neutrophils. The portal LPS infusion experiment suggests that damaged LSECs and gut-derived LPS synergistically contribute to hepatic neutrophil recruitment and activation, thus facilitating colitis-induced liver injury.

**FIGURE 8:**
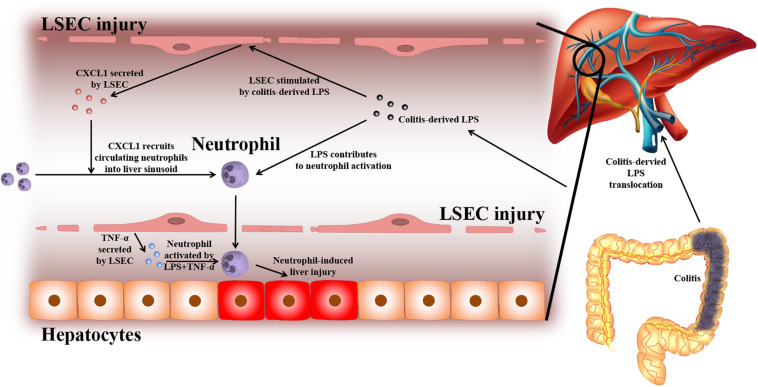
Proposed mechanism of LSEC barrier in the gut–liver axis. A leaky gut can lead to the translocation of gut-derived pathogenic factors [e.g., lipopolysaccharide (LPS)] to the portal vein and encounter liver sinusoidal endothelial cells (LSECs). Under normal conditions, the intact LSEC barrier can defend against these invasive factors. When LSECs are damaged, they can shift toward a pro-inflammatory phenotype. Under the stimulation of gut-derived LPS, damaged LSECs can secrete pro-inflammatory mediators including neutrophil-chemoattractant CXCL1 and TNF-α. CXCL1 induces the recruitment of peripheral blood neutrophils into the liver. Neutrophils are further activated by gut-derived LPS and LSEC-derived TNF-α. Activated neutrophils can produce massive reactive oxygen species (ROS), pro-inflammatory cytokines, and neutrophil extracellular traps (NETs), which lead to enhanced liver injury.

Based on the experiments above, the findings of this study can be summarized as follows (depicted in Figure 8): due to a leaky gut, gut-derived pathogenic factors (e.g., LPS) can translocate to the liver and encounter LSECs. Under normal conditions, the intact barrier of LSECs can protect the liver against gut-derived pathogenic factors. However, when LSECs are damaged, they turn into a pro-inflammatory phenotype. In response to gut-derived LPS, injured LSECs secrete CXCL1 and TNF-α. CXCL1 recruits peripheral neutrophils into the liver. Under the co-stimulation of gut-derived LPS and LSEC-derived TNF-α, hepatic neutrophils are activated and produce ROS, pro-inflammatory cytokines, and NETs, and finally lead to aggravated liver injury.

## Discussion

Currently, most studies in the field of the gut–liver axis have focused on the effect of the gut barrier. A leaky gut may allow the passage of toxins, antigens, and bacteria in the lumen to enter the bloodstream and liver, thus eliciting negative effects on the liver. However, our study demonstrates that there is also a barrier in the liver, defending against gut-derived invasive factors, and LSECs play a key role in this barrier. In several liver diseases with LSEC injury, the barrier function of LSECs can also be damaged. As a consequence, exposure to colitis-derived injurious factors (e.g., LPS) will lead to aberrant immune activation and significant liver injury. Therefore, an intact sinusoidal endothelial is of vital importance in protecting the liver from colitis-induced injury.

Based on the hypothesis in this study, we thoroughly explored the hepatic barrier by using various liver disease models, and many other liver cells were also investigated including macrophages, B cells, CD4^+^ T cells, and CD8^+^ T cells. We found that these cells were substantially unchanged in colitis-induced liver injuries. Eventually, LSECs were identified to possess barrier function toward gut-derived invasive factors. In terms of anatomical features, LSECs are the first-line liver cells confronted with portal-delivered gut-derived antigens. It is a potential antigen-presenting cell due to the expression of costimulatory molecules, such as CD40, CD80, and CD86 (Crispe, 2009). LSECs usually regulate the local immunity toward tolerance rather than activation (Xu et al., 2016). For instance, under normal conditions, LSECs respond to LPS by secreting immunosuppressive IL-10 (Crispe, 2009). LSECs also express a scavenger receptor, which renders LSECs able to take in certain pathogens (Mates et al., 2017; Oie et al., 2020) and circulating metabolites (Wang and Liu, 2021a). Since the gut contains a huge population of bacteria, the level of LPS in the portal vein that collects the intestinal blood was reported to be higher (Lumsden et al., 1988). When the gut barrier is injured, the LPS level can be further increased (Guo et al., 2015). LSECs are particularly capable of removing LPS (Suzuki et al., 2016; Yao et al., 2016). Whether the barrier function of LSECs can resist gut-derived injurious factors is largely unknown, because most previous studies only investigated the damage of sinusoidal endothelial cells in liver diseases. However, we have found that when LSECs are damaged, DSS colitis can more easily induce liver injury. When injured by MCT *ex vivo*, LSECs shift toward a pro-inflammatory pattern under LPS stimulation, with elevated CXCL1 and TNF-α expression. The mechanism of such phenotype alteration of LSECs remains unclear. Several signaling pathways have been reported to be involved in the pro-inflammatory phenotype of LSECs, including the downregulation of vascular endothelial growth factor-stimulated NO production (Deleve et al., 2008), NOX1 upregulation in certain liver diseases, such as NAFLD (Matsumoto et al., 2018), and decreased nitric oxide synthase 3 expression (Takada et al., 2018). Future studies need to further clarify the mechanisms of phenotype reversal of damaged LSECs.

LSEC injury in different liver diseases is usually characterized by various features, including the loss of fenestrae, detachment or defect of the endothelial lining, formation of large gaps, and the formation of basement membrane (Wisse et al., 1985; DeLeve et al., 1999; Wang and Liu, 2021a). In our study, the features of LSEC damage in SOS, LF, and NASH models were quite alike, mainly manifested as the loss of fenestrae, detachment of endothelial lining, and the formation of large gaps, while there was no obvious formation of basement membrane in these models. Yet, the SOS model seemed to induce more critical LSEC injury since the large defect of endothelial lining was observed. Therefore, the different injurious factors in SOS, LF, and NASH models might cause a similar pattern of LSEC damage with variable severity. The damage of LSECs also led to immunological phenotype changes, from an immuno-tolerant to a pro-inflammatory pattern. Several pieces of literature also suggested that the pro-inflammatory effects of LSECs might be associated with the LSEC injury process in several types of liver diseases, such as NAFLD and primary sclerosing cholangitis. In NAFLD, LSECs contributed to the activation of Kupffer cells or expressed IL-6, platelet endothelial cell adhesion molecule-1, and intercellular adhesion molecule-1 (Miyao et al., 2015; Wang and Liu, 2021a). In patients with IBD + primary sclerosing cholangitis, LSECs expressed a high level of mucosal address in cell adhesion molecule-1, which promoted the adhesion of α_4_β_7_^+^ mucosal lymphocytes with hepatic sinusoid endothelium (Grant et al., 2001). Meanwhile in our study, injured LSECs mainly secreted pro-inflammatory CXCL1 and TNF-α under the stimulation of gut-derived LPS. Therefore, LSEC is more than a physical barrier in the gut–liver axis. The damage of LSECs can be caused by a variety of invasive factors, and the destruction of its physical structure is usually accompanied by immunological function changes. Dysfunctional LSECs can lead to the imbalance of the hepatic immune environment and consequences of inflammatory reaction, which is, in our study, the recruitment and activation of hepatic neutrophils.

Recently, increasing attention has been paid to neutrophil-mediated liver injury in various types of liver diseases (Wang and Liu, 2021b). In colitis-aggravated liver injury, we observed elevated neutrophils in the liver as well as in the peripheral blood. Although peripheral blood neutrophils can be increased in various clinical diseases, it rarely leads to liver injury. So how neutrophils enter the liver and induce liver injury is a critical issue. As is known, the recruitment and activation of neutrophils are two key steps in neutrophil-mediated liver injury (Khanam et al., 2017; Wang and Liu, 2021b). Thus, we focus on the two key steps and use the LPS-induced pneumonia model that also has elevated peripheral blood neutrophils. LSECs show a barrier effect since the elevated peripheral blood neutrophils in colitis and pneumonia cannot enter the liver when LSECs are normal. Normal LSECs are immunologically tolerant to colitis-derived LPS, which has also been found in other studies (Uhrig et al., 2005; Suzuki et al., 2016). These peripheral blood neutrophils are mainly recruited to the inflammatory lesions in the lung or colon with the navigation of CXCL1. However, when the LSEC barrier is damaged, colitis-derived LPS can enter the liver sinusoids and lead to elevated CXCL1 expression by LSECs. Therefore, the peripheral neutrophils can be recruited into the liver and induce liver injury. Compared with the gut–liver axis, the lung is less closely linked to the liver due to its anatomical features. The concentration of pneumonia-derived LPS is low in the portal vein. Without the stimulation by LPS, damaged LSECs barely produce CXCL1. Despite damaged LSECs and increased peripheral neutrophils in the pneumonia model, these neutrophils tend to migrate to the lung lesions rather than the liver. Therefore, LPS, especially derived from the gut, is a key trigger for the recruitment of peripheral blood neutrophils into the liver.

In *ex vivo* experiments, we found that neutrophils in colitis and pneumonia models could be activated by LSECs. Thus, we further used the HCl-induced pneumonia model that was not associated with the effect of LPS and proved that LPS enabled neutrophils to be activated. As is known, LPS is a common stimulus to neutrophil priming (El-Benna et al., 2016). In resting normal circulating neutrophils, the microbicidal capacity and cytotoxicity are very low even when exposed to activating stimuli. LPS can prime neutrophils *via* multiple ways, such as inducing the expression of the fMLF receptor at the plasma membrane, cytochrome b_558_ mobilization, and the translocation of p47^phox^ to the membrane (El-Benna et al., 2016). Once primed, neutrophils can produce enhanced respiratory bursts and induce cytotoxicity when triggered by a secondary activating stimulus. In our study, LPS might be responsible for neutrophils priming in the colitis model *in vivo*; thus, neutrophils can further be activated within the liver, while for those primed neutrophils in the pneumonia model, they are recruited into the lung instead of the liver due to the concentration gradient of CXCL1. As a result, despite increased peripheral neutrophils in pneumonia patients, liver injury rarely occurs.

Our study suggests that LSECs participate in both the recruitment and activation of neutrophils by secreting CXCL1 and TNF-α, respectively. The interactions between hepatic neutrophils and LSECs have also been reported in other studies (McDonald et al., 2013). Recently, Yazdani et al. (2017) have found that LSECs are capable of releasing IL-33, which can trigger neutrophil activation through its receptor ST2. However, in our study, LSECs undergoing different treatments showed no difference in the expression of IL-33. Instead, we observed enhanced secretion of TNF-α by LSECs. TNF-α has also been well-proven to be a potent activator for neutrophils in previous studies (Zou et al., 2011). We also observed massive production of ROS and the formation of NETs by activated neutrophils. The oxidative stress injury of hepatocytes by ROS has been implied by the elevated level of MDA. As for NETs, in recent years, a substantial body of evidence has emerged, showing that NETs participate in the pathophysiology of sterile inflammatory reactions in several liver diseases, such as hepatocellular carcinoma, portal hypertension, and ischemia/reperfusion injury (Huang et al., 2015; Kolaczkowska et al., 2015; Sakurai et al., 2017; van der Windt et al., 2018; Hilscher et al., 2019). Arumugam et al. (2018) reported that NETs could lead to nuclear DNA damage and the loss of mitochondrial integrity of hepatocytes *in vitro*. In clinical cases, selective leukocytapheresis has been used in the treatment of ulcerative colitis and shows a promising effect on improving response and remission rates (Zhu et al., 2011). Therefore, the effect of neutrophils is vitally important in the occurrence and development of liver injury. The mechanism of colitis-induced liver injury is rather different from other types of liver injury.

## Conclusion

Through different types of liver disease models, we have demonstrated that the LSEC barrier plays a key role in protecting the liver against gut-derived invasive factors. By using liver injury, pneumonia, and colitis models, we further prove that when the LSEC barrier is damaged, gut-derived LPS can lead to the recruitment and activation of hepatic neutrophils. The neutrophil is an important downstream effector in colitis-induced liver injury *via* producing ROS, pro-inflammatory cytokines, and NETs. Our study draws particular attention to the importance of LSECs in clinical cases of liver disease, especially in patients complicated with gut disorders. The hepatotoxic mechanism of neutrophils in colitis-induced liver injury should be further elucidated.

## Data Availability Statement

The original contributions presented in the study are included in the article/Supplementary Material, further inquiries can be directed to the corresponding author/s.

## Ethics Statement

The animal study was reviewed and approved by the Ethics Committee of Peking University People’s Hospital.

## Author Contributions

YW and YlL proposed the study concept and design, contributed to the interpretation of data, and drafted the manuscript. YW, YZ, and YnL performed the experiments. YW, JX, and YlL discussed the significance of this research. YW and YnL collected the data and performed the data analysis. YlL critically revised the manuscript. All authors contributed to manuscript revision, and read and approved the submitted version.

## Conflict of Interest

The authors declare that the research was conducted in the absence of any commercial or financial relationships that could be construed as a potential conflict of interest.

## Supplementary Material

Supplementary Table 1 The primers used in PCR reaction.

Supplementary Table 2 Disease activity index score.

Supplementary Materials and Methods More detailed methods of techniques including electron microscope, H&E staining, ELISA, flow cytometry, immunohistochemistry, immunocytochemistry, PCR, Western blot, and Transwell experiment were also described.

## Abbreviations

IBD: inflammatory bowel disease
NAFLD: non-alcoholic fatty liver disease
DILI: drug-induced liver injury
DSS: dextran sulfate sodium
LPS: lipopolysaccharide
NASH: non-alcoholic steatohepatitis
LSECs: liver sinusoidal endothelial cells
SOS: sinusoidal obstruction syndrome
MCT: monocrotaline
CCl_4_: carbon tetrachloride
ConA: Concanavalin-A
ALT: aminotransferase
AST: aspartate aminotransferase
SEM: scanning electron microscopy
TEM: transmission electron microscopy
IHC: immunohistochemistry
MPO: myeloperoxidase
ROS: reactive oxygen species
MDA: malondialdehyde
NETs: neutrophil extracellular traps
RECA1: rat endothelial cell antibody-1.

## References

[B1] Achiwa K. Ishigami M. Ishizu Y. Kuzuya T. Honda T. Hayashi K. (2016). DSS colitis promotes tumorigenesis and fibrogenesis in a choline-deficient high-fat diet-induced NASH mouse model. *Biochem. Biophys. Res. Commun.* 470 15–21. 10.1016/j.bbrc.2015.12.012 26682925

[B2] Arumugam S. Girish Subbiah K. Kemparaju K. Thirunavukkarasu C. (2018). Neutrophil extracellular traps in acrolein promoted hepatic ischemia reperfusion injury: therapeutic potential of NOX2 and p38MAPK inhibitors. *J. Cell. Physiol.* 233 3244–3261. 10.1002/jcp.26167 28884828

[B3] Bleau C. Filliol A. Samson M. Lamontagne L. (2016). Mouse hepatitis virus infection induces a toll-like receptor 2-dependent activation of inflammatory functions in liver sinusoidal endothelial cells during acute hepatitis. *J. Virol.* 90 9096–9113. 10.1128/JVI.01069-16 27489277 PMC5044860

[B4] Braet F. De Zanger R. Sasaoki T. Baekeland M. Janssens P. Smedsrod B. (1994). Assessment of a method of isolation, purification, and cultivation of rat liver sinusoidal endothelial cells. *Lab. Invest.* 70 944–952. 8015298

[B5] Conway R. Low C. Coughlan R. J. O’Donnell M. J. Carey J. J. (2015). Risk of liver injury among methotrexate users: a meta-analysis of randomised controlled trials. *Semin. Arthritis Rheum.* 45 156–162. 10.1016/j.semarthrit.2015.05.003 26088004

[B6] Crispe I. N. (2009). The liver as a lymphoid organ. *Annu. Rev. Immunol.* 27 147–163. 10.1146/annurev.immunol.021908.132629 19302037

[B7] DeLeve L. D. McCuskey R. S. Wang X. Hu L. McCuskey M. K. Epstein R. B. (1999). Characterization of a reproducible rat model of hepatic veno-occlusive disease. *Hepatology* 29 1779–1791. 10.1002/hep.510290615 10347121

[B8] Deleve L. D. Wang X. Guo Y. (2008). Sinusoidal endothelial cells prevent rat stellate cell activation and promote reversion to quiescence. *Hepatology* 48 920–930. 10.1002/hep.22351 18613151 PMC2695448

[B9] El Kasmi K. C. Anderson A. L. Devereaux M. W. Fillon S. A. Harris J. K. Lovell M. A. (2012). Toll-like receptor 4-dependent Kupffer cell activation and liver injury in a novel mouse model of parenteral nutrition and intestinal injury. *Hepatology* 55 1518–1528. 10.1002/hep.25500 22120983 PMC4986925

[B10] El-Benna J. Hurtado-Nedelec M. Marzaioli V. Marie J. C. Gougerot-Pocidalo M. A. Dang P. M. (2016). Priming of the neutrophil respiratory burst: role in host defense and inflammation. *Immunol. Rev.* 273 180–193. 10.1111/imr.12447 27558335

[B11] Feagan B. G. McDonald J. W. Panaccione R. Enns R. A. Bernstein C. N. Ponich T. P. (2014). Methotrexate in combination with infliximab is no more effective than infliximab alone in patients with Crohn’s disease. *Gastroenterology* 146 681–688. 10.1053/j.gastro.2013.11.024 24269926

[B12] Fournier M. R. Klein J. Minuk G. Y. Bernstein C. N. (2010). Changes in liver biochemistry during methotrexate use for inflammatory bowel disease. *Am. J. Gastroenterol.* 105 1620–1626. 10.1038/ajg.2010.21 20160715

[B13] Gabele E. Dostert K. Hofmann C. Wiest R. Scholmerich J. Hellerbrand C. (2011). DSS induced colitis increases portal LPS levels and enhances hepatic inflammation and fibrogenesis in experimental NASH. *J. Hepatol.* 55 1391–1399. 10.1016/j.jhep.2011.02.035 21703208

[B14] Gisbert J. P. Luna M. Gonzalez-Lama Y. Pousa I. D. Velasco M. Moreno-Otero R. (2007). Liver injury in inflammatory bowel disease: long-term follow-up study of 786 patients. *Inflamm. Bowel Dis.* 13 1106–1114. 10.1002/ibd.20160 17455203

[B15] Grant A. J. Lalor P. F. Hubscher S. G. Briskin M. Adams D. H. (2001). MAdCAM-1 expressed in chronic inflammatory liver disease supports mucosal lymphocyte adhesion to hepatic endothelium (MAdCAM-1 in chronic inflammatory liver disease). *Hepatology* 33 1065–1072. 10.1053/jhep.2001.24231 11343233

[B16] Guo Y. Zhou G. He C. Yang W. He Z. Liu Z. (2015). Serum levels of lipopolysaccharide and 1,3-beta-D-glucan refer to the severity in patients with Crohn’s disease. *Mediators Inflamm.* 2015:843089. 10.1155/2015/843089 26106258 PMC4464677

[B17] Hilscher M. B. Sehrawat T. Arab J. P. Zeng Z. Gao J. Liu M. (2019). Mechanical stretch increases expression of CXCL1 in liver sinusoidal endothelial cells to recruit neutrophils, generate sinusoidal microthombi, and promote portal hypertension. *Gastroenterology* 157 193–209. 10.1053/j.gastro.2019.03.013 30872106 PMC6581607

[B18] Huang H. Tohme S. Al-Khafaji A. B. Tai S. Loughran P. Chen L. (2015). Damage-associated molecular pattern-activated neutrophil extracellular trap exacerbates sterile inflammatory liver injury. *Hepatology* 62 600–614. 10.1002/hep.27841 25855125 PMC4515210

[B19] Khanam A. Trehanpati N. Riese P. Rastogi A. Guzman C. A. Sarin S. K. (2017). Blockade of neutrophil’s chemokine receptors CXCR1/2 abrogate liver damage in acute-on-chronic liver failure. *Front. Immunol.* 8:464. 10.3389/fimmu.2017.00464 28484461 PMC5401894

[B20] Kolaczkowska E. Jenne C. N. Surewaard B. G. Thanabalasuriar A. Lee W. Y. Sanz M. J. (2015). Molecular mechanisms of NET formation and degradation revealed by intravital imaging in the liver vasculature. *Nat. Commun.* 6:6673. 10.1038/ncomms7673 25809117 PMC4389265

[B21] Lumsden A. B. Henderson J. M. Kutner M. H. (1988). Endotoxin levels measured by a chromogenic assay in portal, hepatic and peripheral venous blood in patients with cirrhosis. *Hepatology* 8 232–236. 10.1002/hep.1840080207 3281884

[B22] Marshall J. C. (1998). The gut as a potential trigger of exercise-induced inflammatory responses. *Can. J. Physiol. Pharmacol.* 76 479–484. 10.1139/cjpp-76-5-479 9839072

[B23] Mates J. M. Yao Z. Cheplowitz A. M. Suer O. Phillips G. S. Kwiek J. J. (2017). Mouse liver sinusoidal endothelium eliminates HIV-like particles from blood at a rate of 100 million per minute by a second-order kinetic process. *Front. Immunol.* 8:35. 10.3389/fimmu.2017.00035 28167948 PMC5256111

[B24] Matsumoto M. Zhang J. Zhang X. Liu J. Jiang J. X. Yamaguchi K. (2018). The NOX1 isoform of NADPH oxidase is involved in dysfunction of liver sinusoids in nonalcoholic fatty liver disease. *Free Radic. Biol. Med.* 115 412–420. 10.1016/j.freeradbiomed.2017.12.019 29274380 PMC5969997

[B25] McDonald B. Jenne C. N. Zhuo L. Kimata K. Kubes P. (2013). Kupffer cells and activation of endothelial TLR4 coordinate neutrophil adhesion within liver sinusoids during endotoxemia. *Am. J. Physiol. Gastrointest. Liver Physiol.* 305 G797–G806. 10.1152/ajpgi.00058.2013 24113769

[B26] Miyao M. Kotani H. Ishida T. Kawai C. Manabe S. Abiru H. (2015). Pivotal role of liver sinusoidal endothelial cells in NAFLD/NASH progression. *Lab. Invest.* 95 1130–1144. 10.1038/labinvest.2015.95 26214582

[B27] Ohtani N. Kawada N. (2019). Role of the gut-liver axis in liver inflammation, fibrosis, and cancer: a special focus on the gut microbiota relationship. *Hepatol. Commun.* 3 456–470. 10.1002/hep4.1331 30976737 PMC6442695

[B28] Oie C. I. Wolfson D. L. Yasunori T. Dumitriu G. Sorensen K. K. McCourt P. A. (2020). Liver sinusoidal endothelial cells contribute to the uptake and degradation of entero bacterial viruses. *Sci. Rep.* 10:898. 10.1038/s41598-020-57652-0 31965000 PMC6972739

[B29] Sakurai K. Miyashita T. Okazaki M. Yamaguchi T. Ohbatake Y. Nakanuma S. (2017). Role for neutrophil extracellular traps (NETs) and platelet aggregation in early sepsis-induced hepatic dysfunction. *In Vivo* 31 1051–1058. 10.21873/invivo.11169 29102925 PMC5756631

[B30] Seo Y. S. Shah V. H. (2012). The role of gut-liver axis in the pathogenesis of liver cirrhosis and portal hypertension. *Clin. Mol. Hepatol.* 18 337–346. 10.3350/cmh.2012.18.4.337 23323248 PMC3540369

[B31] Suzuki K. Murakami T. Hu Z. Tamura H. Kuwahara-Arai K. Iba T. (2016). Human host defense cathelicidin peptide LL-37 enhances the lipopolysaccharide uptake by liver sinusoidal endothelial cells without cell activation. *J. Immunol.* 196 1338–1347. 10.4049/jimmunol.1403203 26729811

[B32] Takada S. Miyashita T. Yamamoto Y. Kanou S. Munesue S. Ohbatake Y. (2018). Soluble thrombomodulin attenuates endothelial cell damage in hepatic sinusoidal obstruction syndrome. *In Vivo* 32 1409–1417. 10.21873/invivo.11393 30348695 PMC6365749

[B33] Thin L. W. Lawrance I. C. Spilsbury K. Kava J. Olynyk J. K. (2014). Detection of liver injury in IBD using transient elastography. *J. Crohns Colitis* 8 671–677. 10.1016/j.crohns.2013.12.006 24529605

[B34] Thomson A. W. Knolle P. A. (2010). Antigen-presenting cell function in the tolerogenic liver environment. *Nat. Rev. Immunol.* 10 753–766. 10.1038/nri2858 20972472

[B35] Uhrig A. Banafsche R. Kremer M. Hegenbarth S. Hamann A. Neurath M. (2005). Development and functional consequences of LPS tolerance in sinusoidal endothelial cells of the liver. *J. Leukoc. Biol.* 77 626–633. 10.1189/jlb.0604332 15860798

[B36] van der Windt D. J. Sud V. Zhang H. Varley P. R. Goswami J. Yazdani H. O. (2018). Neutrophil extracellular traps promote inflammation and development of hepatocellular carcinoma in nonalcoholic steatohepatitis. *Hepatology* 68 1347–1360. 10.1002/hep.29914 29631332 PMC6173613

[B37] Wang Y. Liu Y. (2021a). Gut-liver-axis: barrier function of liver sinusoidal endothelial cell. *J. Gastroenterol. Hepatol*. 10.1111/jgh.15512 [Epub ahead of print]. 33811372

[B38] Wang Y. Liu Y. (2021b). Neutrophil-induced liver injury and interactions between neutrophils and liver sinusoidal endothelial cells. *Inflammation* 10.1007/s10753-021-01442-x [Epub ahead of print]. 33649876

[B39] Wiest R. Albillos A. Trauner M. Bajaj J. S. Jalan R. (2017). Targeting the gut-liver axis in liver disease. *J. Hepatol.* 67 1084–1103. 10.1016/j.jhep.2017.05.007 28526488

[B40] Wisse E. De Zanger R. B. Charels K. Van Der Smissen P. McCuskey R. S. (1985). The liver sieve: considerations concerning the structure and function of endothelial fenestrae, the sinusoidal wall and the space of Disse. *Hepatology* 5 683–692. 10.1002/hep.1840050427 3926620

[B41] Xing Y. Zhao T. Gao X. Wu Y. (2016). Liver X receptor alpha is essential for the capillarization of liver sinusoidal endothelial cells in liver injury. *Sci. Rep.* 6:21309. 10.1038/srep21309 26887957 PMC4758044

[B42] Xu X. Jin R. Li M. Wang K. Zhang S. Hao J. (2016). Liver sinusoidal endothelial cells induce tolerance of autoreactive CD4+ recent thymic emigrants. *Sci. Rep.* 6:19861. 10.1038/srep19861 26794038 PMC4726350

[B43] Yao Z. Mates J. M. Cheplowitz A. M. Hammer L. P. Maiseyeu A. Phillips G. S. (2016). Blood-borne lipopolysaccharide is rapidly eliminated by liver sinusoidal endothelial cells via high-density lipoprotein. *J. Immunol.* 197 2390–2399. 10.4049/jimmunol.1600702 27534554 PMC5010928

[B44] Yazdani H. O. Chen H. W. Tohme S. Tai S. van der Windt D. J. Loughran P. (2017). IL-33 exacerbates liver sterile inflammation by amplifying neutrophil extracellular trap formation. *J. Hepatol*. 68 130–139. 10.1016/j.jhep.2017.09.010 28943296 PMC5862757

[B45] Zhu M. Xu X. Nie F. Tong J. Xiao S. Ran Z. (2011). The efficacy and safety of selective leukocytapheresis in the treatment of ulcerative colitis: a meta-analysis. *Int. J. Colorectal Dis.* 26 999–1007. 10.1007/s00384-011-1193-9 21476027

[B46] Zou W. Roth R. A. Younis H. S. Malle E. Ganey P. E. (2011). Neutrophil-cytokine interactions in a rat model of sulindac-induced idiosyncratic liver injury. *Toxicology* 290 278–285. 10.1016/j.tox.2011.10.005 22019926 PMC3226905

