# Supplementary material for: Gut–Liver Axis: Liver Sinusoidal Endothelial Cells Function as the Hepatic Barrier in Colitis-Induced Liver Injury
Source: Front Cell Dev Biol. 2021 Jul 16;9:702890. doi: 10.3389/fcell.2021.702890 (PMC8322652; doi:10.3389/fcell.2021.702890)
Supplement: Supplementary Table 1 — The primers used in PCR reaction. [file Data_Sheet_1.PDF]

Supplementary Materials and Methods

SEM and TEM

Rat was anesthetized, and perfusion with HBSS followed by electron microscope fixative buffer (Servicebio) via the portal vein was performed. The perfusion was stopped until surface of the liver became hard and tough. Liver samples were cut into appropriate size and shape for SEM (JSM-7900F, JEOL) and TEM (JEOL) testing.

H&E staining

Liver tissue fixed in neutral buffered formalin was embedded in paraffin. Tissue was sectioned into slices at 4 μm thickness, then baked at 60℃for 4 h. After paraffin was removed by xylene and a graded ethanol series, sections were stained with hematoxylin and eosin.

Whole blood cell test

Whole blood cell test was performed using an automatic animal blood cell analyzer (BC-2800vet, Mindray).

Biomarkers for liver injury

After centrifugation of blood samples for 10 min at 3,300 rpm, serum were collected. The activities of serum alanine aminotransferase (ALT) and aspartate transaminase (AST) were measured with ALT and AST assay reagent kits purchased from Nanjing Jiancheng Bioengineering Institute (Nanjing, China) according to the manufacturers' instructions. For evaluation of hepatocyte lipid peroxidation injury, malondialdehyde (MDA) was measured with the kits (Nanjing Jiancheng Bioengineering Institute, China) in accordance with the manufacturers' instructions.

Enzyme-linked immunosorbent assay (ELISA)

Chemokines and cytokines in the serum and cell-culture supernatant were measured using ELISA kits obtained from Multi Sciences Biotech according to the manufacturers' protocol. Briefly, serum/supernatant, standard solutions and detection antibody were added in the 96-well plate pre-coated by capture antibody. Subsequently incubation with streptavidin-HRP solution, substrate solution, then stop solution was performed. Evaluation was performed using a microplate reader with 450 nm wavelength and 630nm wavelength for reference. ELISA kit for lipopolysaccharide was obtained from Cloud-clone.

Flow cytometry

Fresh liver lobes were placed in ice-cold PBS and minced with scissors. Cell suspension was obtained via a 70μm cell strainer. While for lung, lung tissues were minced with scissors and digested with RPMI-1640 containing collagenase IV (0.5 mg/mL) and DNase I (0.02mg/mL, Solarbio). Then nonparenchymal cells were obtained after Percoll density gradient (40% and 80%, SigmaAldrich) centrifugation. Lysis of red blood cells was performed using red blood cell lysis buffer (BioLegend). Then cell-surface markers were stained using the following antibodies on ice for 20 minutes in the dark: anti-CD3 (BioLegend), anti-CD4 (BioLegend), anti-CD8a (BioLegend), anti-CD11b/c (BioLegend) and anti-rat-granulocytes (BD Biosciences). After washed with PBS, cells were fixed with fixation buffer (BioLegend). Then permeabilization was performed using permeabilization wash buffer (BioLegend). Afterwards intracellular marker was stained with anti-CD68 (Bio-Rad) for 20 minutes

in the dark. Samples were measured on Gallios (Beckman).

For detecting neutrophils-derived ROS, cells were washed twice with PBS, then incubated with DCFH-DA (1:2000, Solarbio) and anti-rat-granulocytes (BD Biosciences) for 30 minutes. After washed with PBS for twice, the fluorescence of ROS (green) and neutrophils (red) were tested by flow cytometry.

FACS

For fluorescence activated cell sorting (FACS), the sorting strategy is shown in **Figure S4**. Cell sorting was performed on Aria II SORP (Beckman). For the detection of ROS by flow cytometry, DCFH-DA (1:2000, Solarbio) was used as a fluorescent probe.

Immunohistochemistry

Slides were deparaffinized by xylene and a graded ethanol series, and incubated in 0.3% H_2O_2 in TBS for 15 min for endogenous peroxide blockage. After antigen retrieval, non-specific binding was blocked with 10% goat serum for 30 minutes at room temperature. Slides were washed by PBS and incubated with primary antibody (for detection of neutrophils: anti-myeloperoxidase rabbit antibody, GB11224, 1:1000; macrophage: anti-CD68 rabbit antibody, GB11067, 1:500; B cell: anti-CD19 rabbit antibody, GB11061-1, 1:400; T cell: anti-CD3 rabbit antibody, GB111337, 1:1000; these antibodies were obtained from Servicebio) overnight at 4℃. After incubation with a poly-peroxidase-conjugated goat ant-rabbit IgG (ZSGB-BIO) at 37℃ for 30 minutes, detection was performed using 3,3'-diaminobenzidine (DAB) and counterstained with haematoxylin. After dehydration, slides were mounted with glycerin.

Immunocytochemistry

For detecting CXCL1 expression by LSECs: LSECs on glass coverslips coated by rat tail tendon collagen were washed twice with PBS, and then fixed with ice-cold 4% paraformaldehyde in PBS. The samples were incubated with PBS containing 0.25% Triton X-100 for 10 min and washed by PBS. Cells were then blocked by 5% BSA for 30 min. Afterwards, LSECs were incubated with diluted antibodies (RECA-1, 1:400; CXCL1, 1:100; Abcam) in 5% BSA for 1 h at room temperature. After washed with PBS, LSECs were then incubated with secondary antibodies (goat anti-mouse IgG Alexa Fluor® 488, 1:200; goat anti-rabbit IgG Alexa Fluor® 594, 1:200; ZSGB-BIO) in 5% BSA for 1 h at room temperature in the dark, and washed by PBS. At last, the coverslips were mounted on glass slides by the fluorescent mounting medium with DAPI (ZSGB-BIO).

For detecting neutrophils-derived NETs: live neutrophils were washed twice with PBS, then incubated with SYTOX Green and anti-rat-granulocytes (BD Biosciences) for 30 minutes. After washed with PBS for twice, the fluorescence of extracellular DNA (green) and neutrophils (red) were tested by fluorescent microscopy.

Immunofluorescence staining for liver ROS detection

Frozen liver tissues embedded in optimal cutting temperature compound (OCT, Sakura Finetek) were cut into 7 mm sections. Frozen slides were restored to room temperature and liquid was thrown away gently. Objective tissue was marked with fluid blocker pen. ROS staining solution DHE (dihydroethidium, Servicebio) was added to the marked area. Samples were then incubated at 37℃ for 30 min in dark place. Afterwards, slides were wash three times with PBS for 5 min each. Then nuclei

were counterstained by incubation with DAPI solution at room temperature for 10 min. After washed with PBS for 3 times, 5 min each, slides were coversliped with anti-fade mounting medium (Servicebio). Fluorescent microscopy (ECLIPSE C1, Nikon, Japan) was used for fluorescence detection.

RNA extraction, reverse transcription and real-time polymerase chain reaction (PCR)

Total RNA of tissue and cells was extracted with Trizol reagent (Thermo Fisher Scientific). Equal amounts of total RNA (1μg) was used for reverse transcription by first strand cDNA synthesis kit (Thermo Fisher Scientific) according to manufacturers' protocol.

Primer were synthesized by Sangon Biotech. Sequences of the primers used in PCR reaction was listed in **Table S1**. PCR for β-actin served as control. PCR was performed using Top Green qPCR SuperMix (TransGen Biotech) according to the manufacturers' instruction with 100 ng cDNA as the template. ABI 7500 Real-Time PCR System (Thermo Fisher Scientific) was used for PCR reactions. The reaction conditions consisted of an initial denaturation step at 94 ℃ for 30 s, followed by 45 cycles at 94 ℃ for 5 s, 60 ℃ for 15 s, and 72 ℃ for 10 s. mRNA expression levels were normalized to levels of β-actin mRNA measured in the same RNA sample and evaluated using the Comparative CT method($2^{-\Delta\Delta CT}$).

Table S1. Sequences of the primers used in PCR reaction.

Primer	Sequences
β-actin	5'-CCGCGAGTACAACCTTCTTG-3'
	5'-CAGTTGGTGACAATGCCGTG-3'
IL-1β	5'-CACCTCTCAAGCAGAGCACA-3'
	5'-ACGGGTTCCATGGTGAAGTC-3'
IL-4	5'-CTGTAGAGGTGTCAGCGGTC-3'
	5'-TCATTCACGGTGCAGCTTCT-3'
IL-6	5'-CTGGTCTTCTGGAGTTCCGT-3'
	5'-TGGTCCTTAGCCACTCCTTCT-3'
IL-12	5'-AAGTTCTTCGTCCGCATCCA-3'
	5'-AAGTTCTTCGTCCGCATCCA-3'
IL-33	5'-CCCGCCTTGCAAAATCACAA-3'
	5'-TCGTAGTAACGGAGTAGCACC-3'
CCL3	5'-GCTTCTCCTATGGACGGCAA-3'
	5'-TGCCGGTTTCTCTTGGTCAG-3'
CCL4	5'-CTCCCGGAAGATTCATCGGA-3'
	5'-TGGCTCTTCCTGTCTTGAGTCT-3'
CCL7	5'-AAGCCCTGAAGACAGATGCC-3'
	5'-CCCCTTAGGACCGTAGTCCA-3'
CXCL1	5'-ATGGCGTCTGTCTGGTGAAC-3'
	5'-ACGACCATCGATGAAACGCA-3'
CXCL2	5'-CCCCTTGGTTCAGAGGATCG-3'
	5'-TTCTGCCCGTTGAGGTACAG-3'
CXCL6	5'-GACCCAGAAGCTCCGTTGAT-3'
	5'-GAACCAGCCCTTCTTTCTTGC-3'
CXCL10	5'-TCCACCTCCCTTTACCCAGT-3'
	5'-AGAGCTAGGAGAGCCGTCAT-3'

TNF-α	5'-ACCATGAGCACGGAAAGCAT-3'
	5'-AACTGATGAGAGGGAGCCCA-3'
IFN-γ	5'-GGAACTGGCAAAAGGACGGT-3'
	5'-TCAGGTGCGATTCGATGACA-3'

Western blot

Total protein was obtained by using lysis buffer (Applygen) according to the manufacturers' instructions. Protein concentration was measured by the BCA protein concentration measurement kit (Beyotime). Then 5* loading buffer was add at the ratio of 4:1. The protein solution was denatured at 95 ℃ for 10 minutes. The concentrated gel and separation gel with appropriate concentrations were prepared and loaded. Add appropriate electrophoresis solution for protein electrophoresis. Concentrated gel voltage was 80V, and separation gel voltage was 130V. When the bromophenol blue was about 1cm from the bottom of the glass, electrophoresis was stopped. Protein was transferred to a PVDF film at 220mA for 60 mintues, then the membrane was blocked for 60 minutes at room temperature by using 5% skim milk. The membrane was incubated with appropriate dilutions of primary antibody (p-p38, p38, p-p65, p65 and GAPDH, obtained from Cell Signaling Technology; p-p47 and p47 obtained from Thermofisher) overnight at 4 ℃. After three washes with TBST, the membrane was incubated with conjugated secondary antibody at room temperature for 60min. Then the film was washed three times with TBST, 15 minutes each time. At last, WB imaging was obtained by using ECL reagents. If chemiluminescent western blots need to be reprobed, primary and secondary antibodies were removed by using stripping buffer obtained from Solarbio. Then re-perform immunoblot experiment from the blocking process with 5% skim milk.

Transwell

6.5 mm Transwell with 5.0 μm pore polycarbonate membrane insert (Sigma, CLS3421) was used. Assemble transwell was inserted in the chamber of 24 wells transwell plate which contained 0.7 mL CXCL1 solution (100, 300, 500 and 1000 pg/mL) or supernatant of LSECs (in the LSEC+MCT+LPS group). Neutrophils in 0.3 mL volume were added into the Transwell insert and incubated for 2 hours in the 37 ℃, 5%CO_2 incubator. Then insert was removed from the chamber. The chamber was rinsed gently in PBS several times to remove unattached cells. Cells were fixed by 10% formalin for 10min, and washed with PBS once. Then cells were stained by 0.1% crystal violet for 15 minutes and washed gently several times with tap water to remove excess stain. The cells from unmigrated (top) side was removed by gently scrapping with a wet cotton swab. Migrated cells were then counted under high power microscope.

Disease Activity Index (DAI)

DAI score was the average score of the three indices in **Table S2**.

Table S2. Disease activity index of DSS-induced colitis

Score	Weight loss percentage	Fecal manifestations	Fecal occult blood
0	<1%	Normal	Negative
1	1-5%	Soft stool	
2	5-10%	Mucoid stool	Positive

3	10-20%		
4	>20%	Diarrhea	Gross blood stool

Supplementary Figure and legends

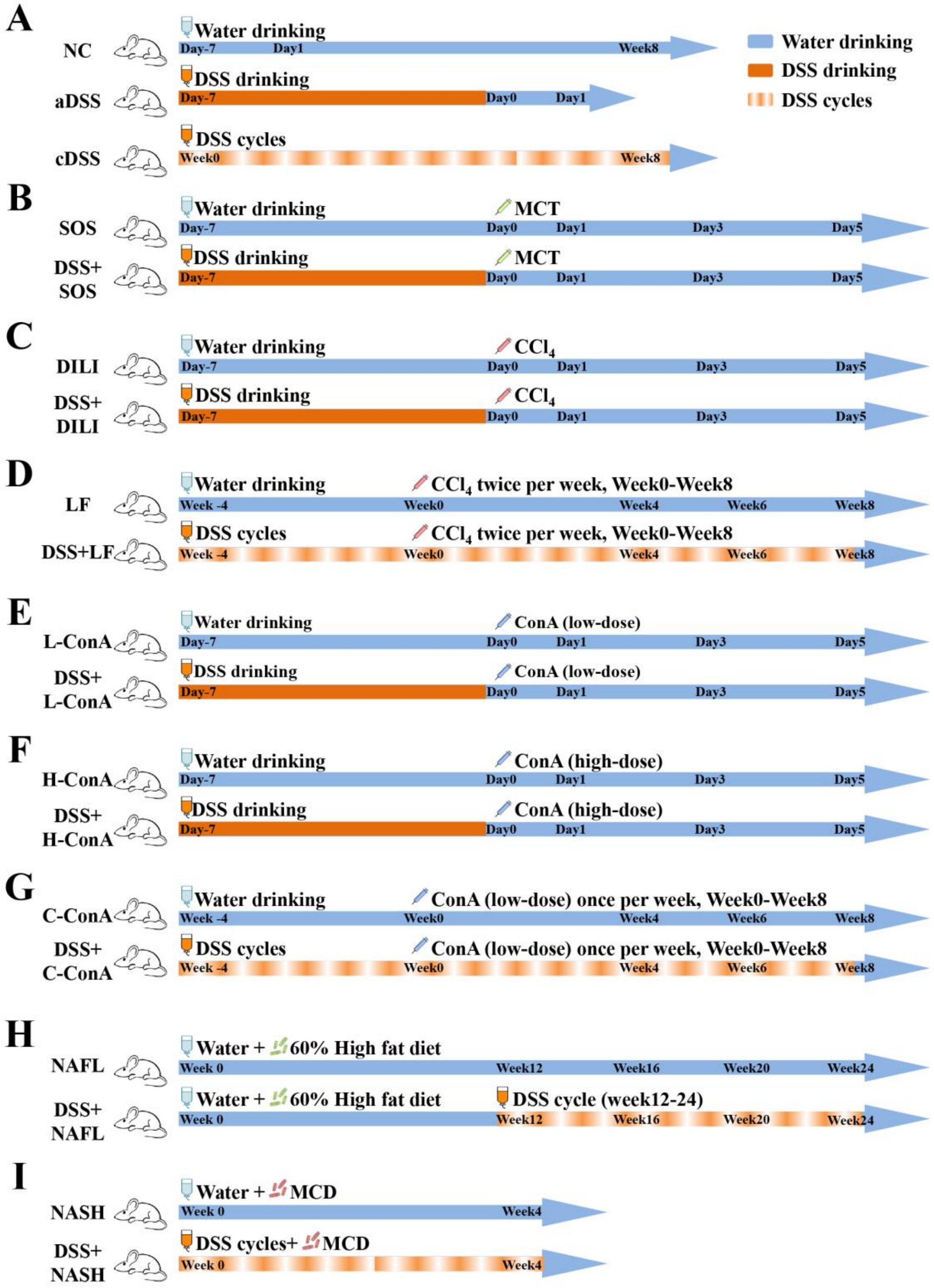

Fig. S1 Animal modeling diagram 1.

To evaluate the effect of dextran sulfate sodium (DSS)-colitis on liver injury, DSS-colitis was induced in combination with the following models: **(A)** normal liver control (NC); **(B)** sinusoidal obstruction syndrome (SOS) induced by monocrotaline (MCT); **(C)** drug-induced liver injury (DILI) model induced by carbon tetrachloride (CCl_4); **(D)** liver fibrosis (LF) model induced by CCl_4; **(E)** low-dose concanavalin-A (ConA) hepatitis model (L-ConA); **(F)** high-dose ConA hepatitis model (H-ConA); **(G)** chronic ConA hepatitis model (C-ConA); **(H)** nonalcoholic fatty liver (NAFL)

model induced by high fat diet; **(I)** nonalcoholic steatohepatitis (NASH) model induced by methionine and choline-deficient diet (MCD).

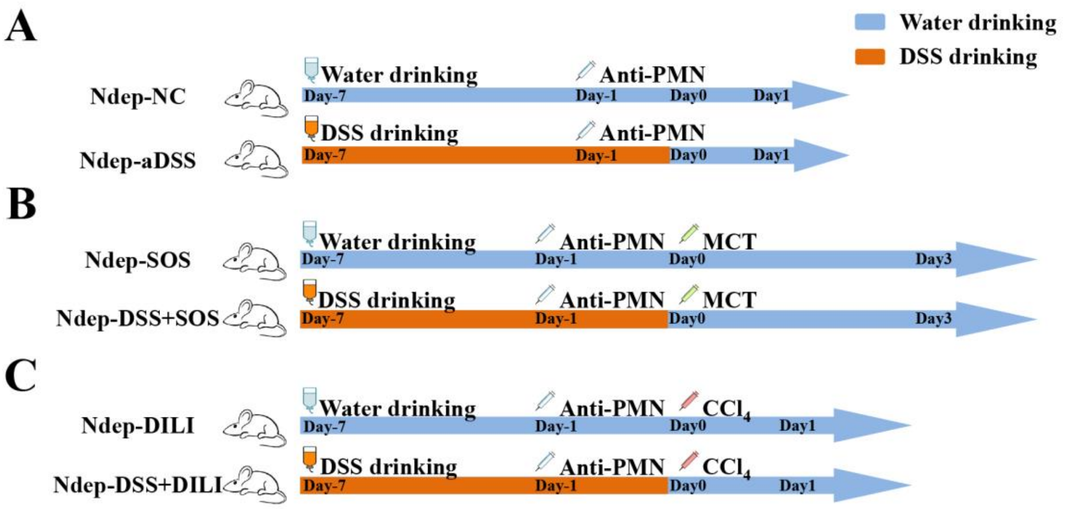

Fig. S2 Animal modeling diagram 2.

To investigate the role of neutrophils in colitis-induced liver injury, anti-polymorphonuclear (PMN) serum was used to induce neutrophils depletion in: **(A)** normal control (Ndep-NC) and acute dextran sulfate sodium-colitis group (Ndep-aDSS); **(B)** sinusoidal obstruction syndrome group (Ndep-SOS) and DSS+SOS group (Ndep-DSS+SOS); **(C)** drug-induced liver injury group (NDep-DILI) and DSS+DILI group (Ddep-DSS+DILI). Abbreviation: MCT, monocrotaline; CCl_4, carbon tetrachloride.

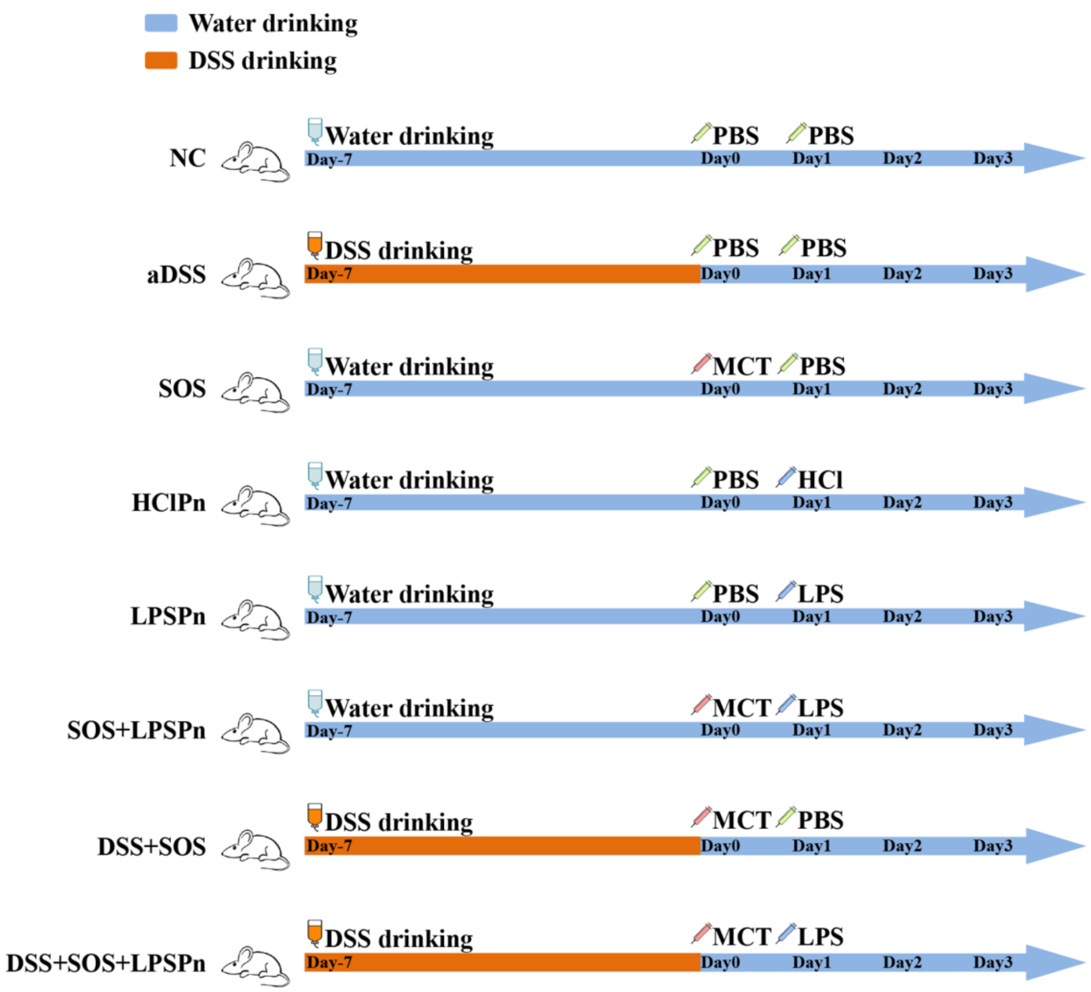

Fig. S3 Animal modeling diagram 3.

To investigate the mechanism of neutrophils recruitment, various disease models were induced, including: normal control (NC), acute dextran sulfate sodium-colitis (aDSS), sinusoidal obstruction syndrome (SOS), HCl-induced pneumonia (HClPn), LPS-induced pneumonia (LPSPn), SOS+LPSPn, DSS+SOS and DSS+SOS+LPSPn. The recruitment of neutrophils and expression of CXCL1 were evaluated at day 3. Abbreviation: PBS, phosphate buffer saline, MCT, monocrotaline;HCl, hydrochloric acid; LPS lipopolysaccharide.

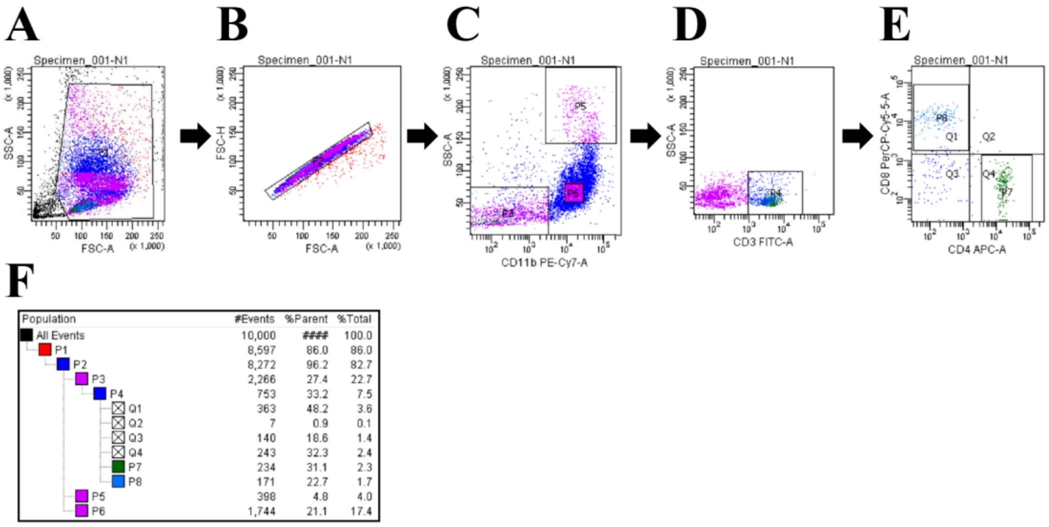

Fig. S4 Sorting strategy of flow cytometry.

Flow cytometry gating for :**(A)** gate P1, complete live cells; **(B)** gate P2, removal of adhesive cells; **(C)** gate P3, lymphocytes; gate P5, neutrophils and gate P6, macrophages; **(D)** gate P4, T lymphocytes; **(E)** gate P7, $CD4^{+}$T cells and gate P8, $CD8^{+}$T cells.

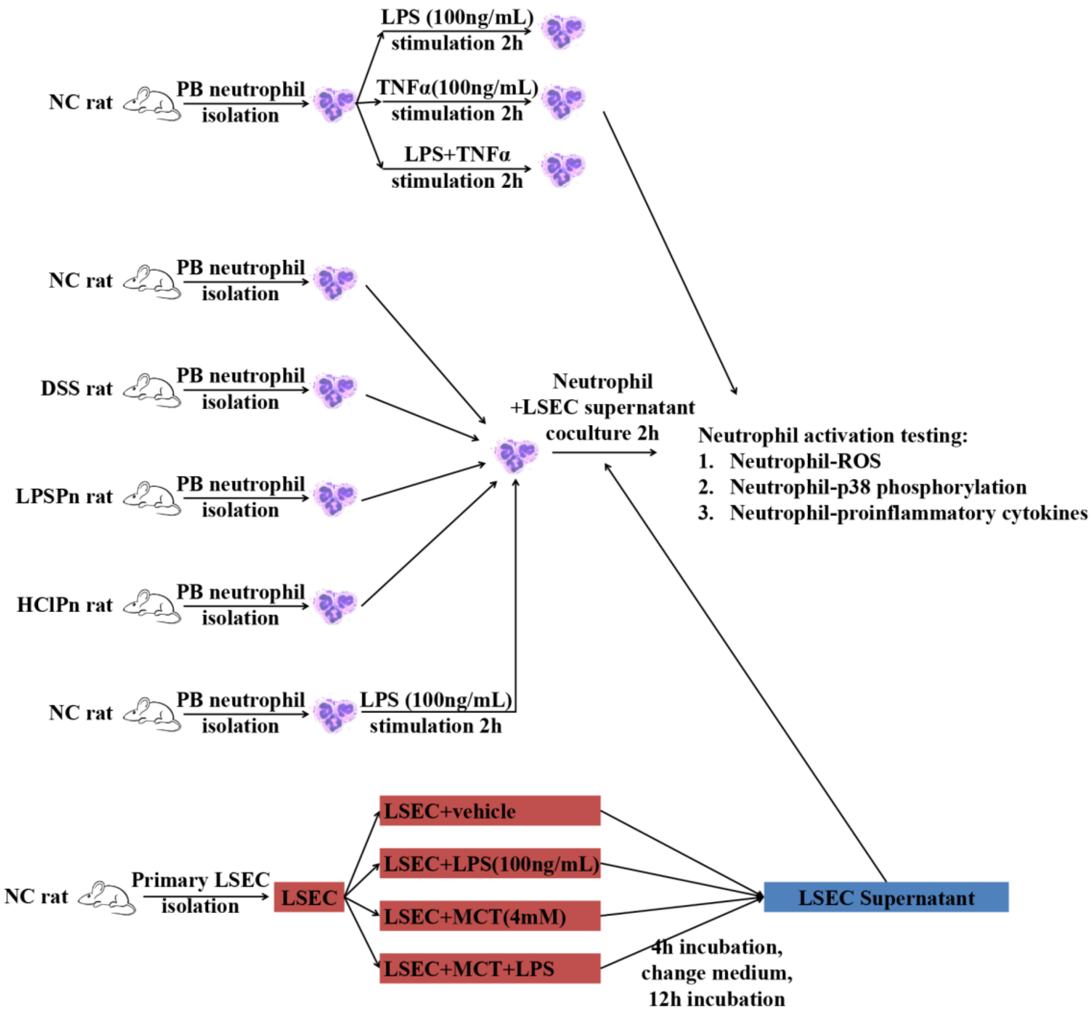

Fig. S5 Experiment flow for investigating the effect of liver sinusoidal endothelial cells (LSECs) on neutrophil activation.

In brief, neutrophils were isolated from the peripheral blood of rats with different diseases, and then incubated with various stimuli (lipopolysaccharide (LPS), TNF-α) or with the supernatant of LSECs undergoing different treatments (vehicle, LPS, monocrotaline (MCT) and MCT+LPS). Then the activation status of neutrophils was evaluated by testing flow cytometry of reactive oxygen species (ROS), phosphorylation of p38 and expression of pro-inflammatory cytokines. Abbreviation: NC, normal control; PB, peripheral blood; DSS, dextran sulfate sodium; LPSPn, LPS-induced pneumonia; HClPn, hydrochloric acid-induced pneumonia.

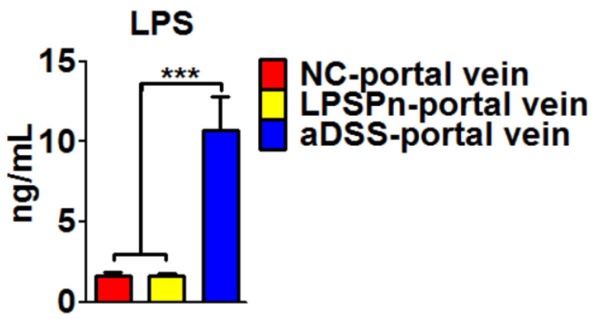

Fig. S6 Lipopolysaccharide concentration in the portal vein blood.

The concentration of lipopolysaccharide (LPS) in the portal vein blood was evaluated in acute normal control group (NC), LPS-induced pneumonia model (LPSPn) and acute dextran sulfate sodium-colitis group (aDSS). *** means P <0.001.

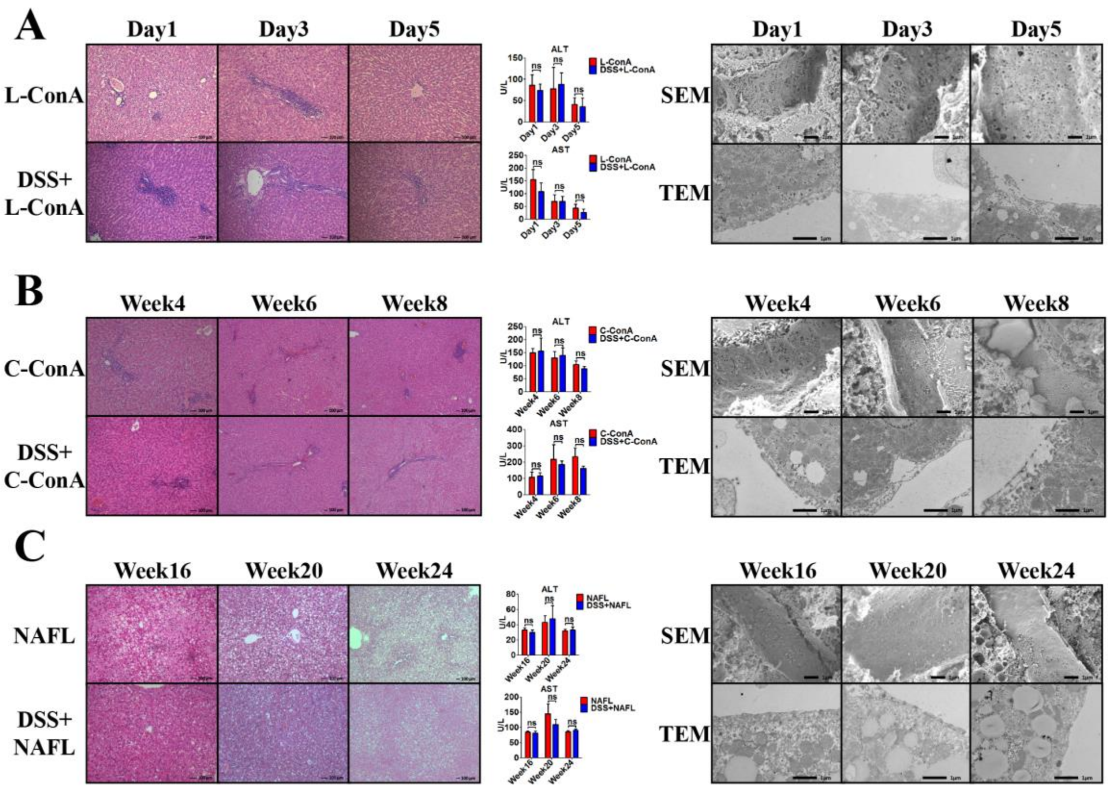

Fig. S7 Effect of colitis on different types of liver injury.

The effect of dextran sulfate sodium (DSS)-colitis on liver injury, and the damage of liver sinusoidal endothelial cells (LSECs) were evaluated in: **(A)** low-dose concanavalin-A (ConA) hepatitis model (L-ConA); **(B)** chronic ConA hepatitis model (C-ConA) and **(C)** nonalcoholic fatty liver (NAFL) model. ns, non-significant; *, *P*<0.05; **, *P*<0.01; ***, *P*<0.001.

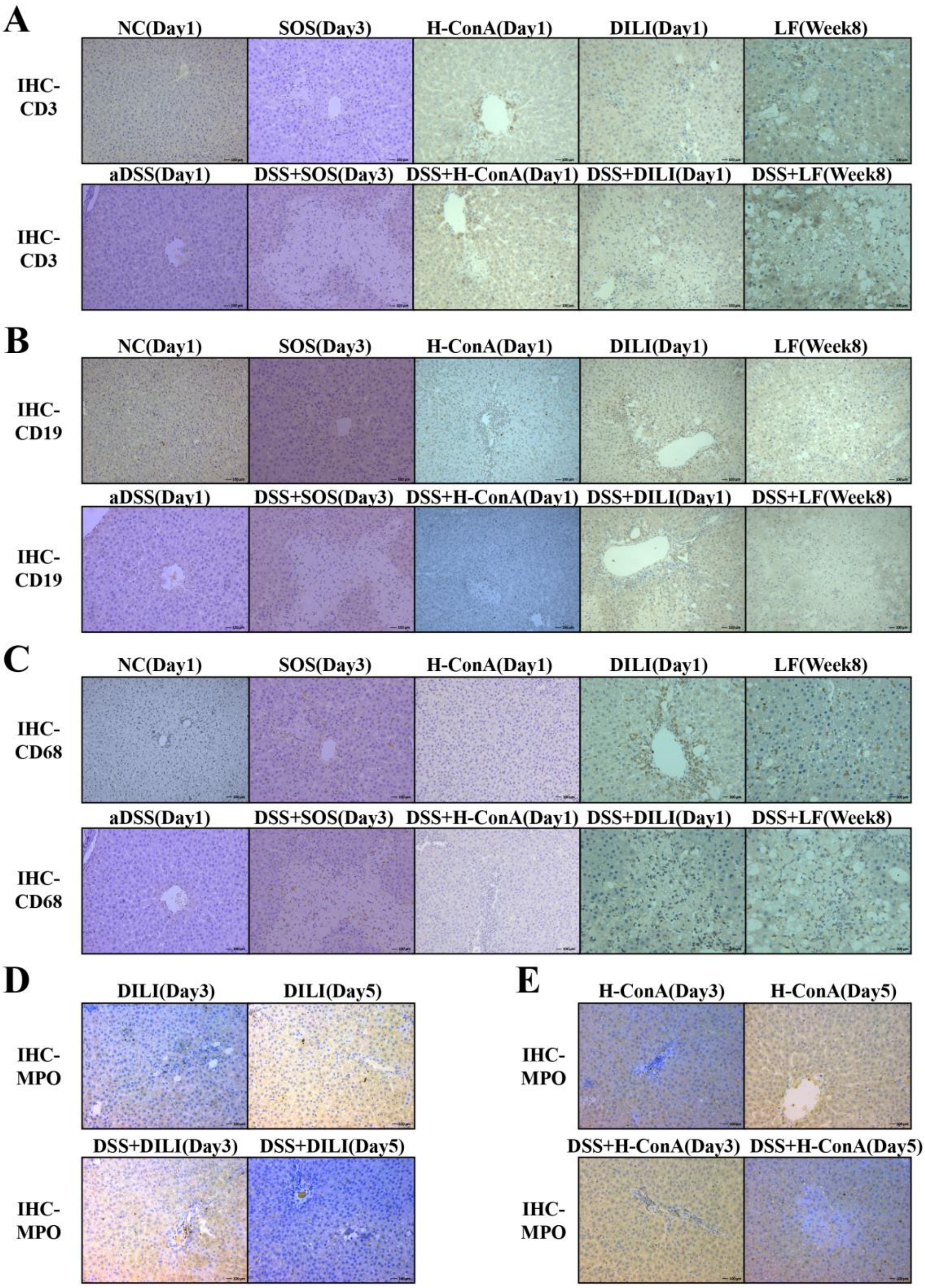

Fig. S8 Evaluation of immunocytes infiltration in colitis-induced liver injury.
The infiltration of **(A)** T cells, **(B)** B cells and **(C)** macrophages in colitis-induced

liver injury were evaluated by immunohistochemistry (IHC) of CD3, CD19 and CD68, respectively. The infiltration of hepatic neutrophils in **(D)** DILI and **(E)** H-ConA models at day3 and day5 were evaluated by IHC of myeloperoxidase (MPO). Abbreviation: NC, normal control; DSS, dextran sulfate sodium-colitis; SOS, sinusoidal obstruction syndrome; H-ConA, high-dose ConA hepatitis; DILI, drug-induced liver injury; LF, liver fibrosis.

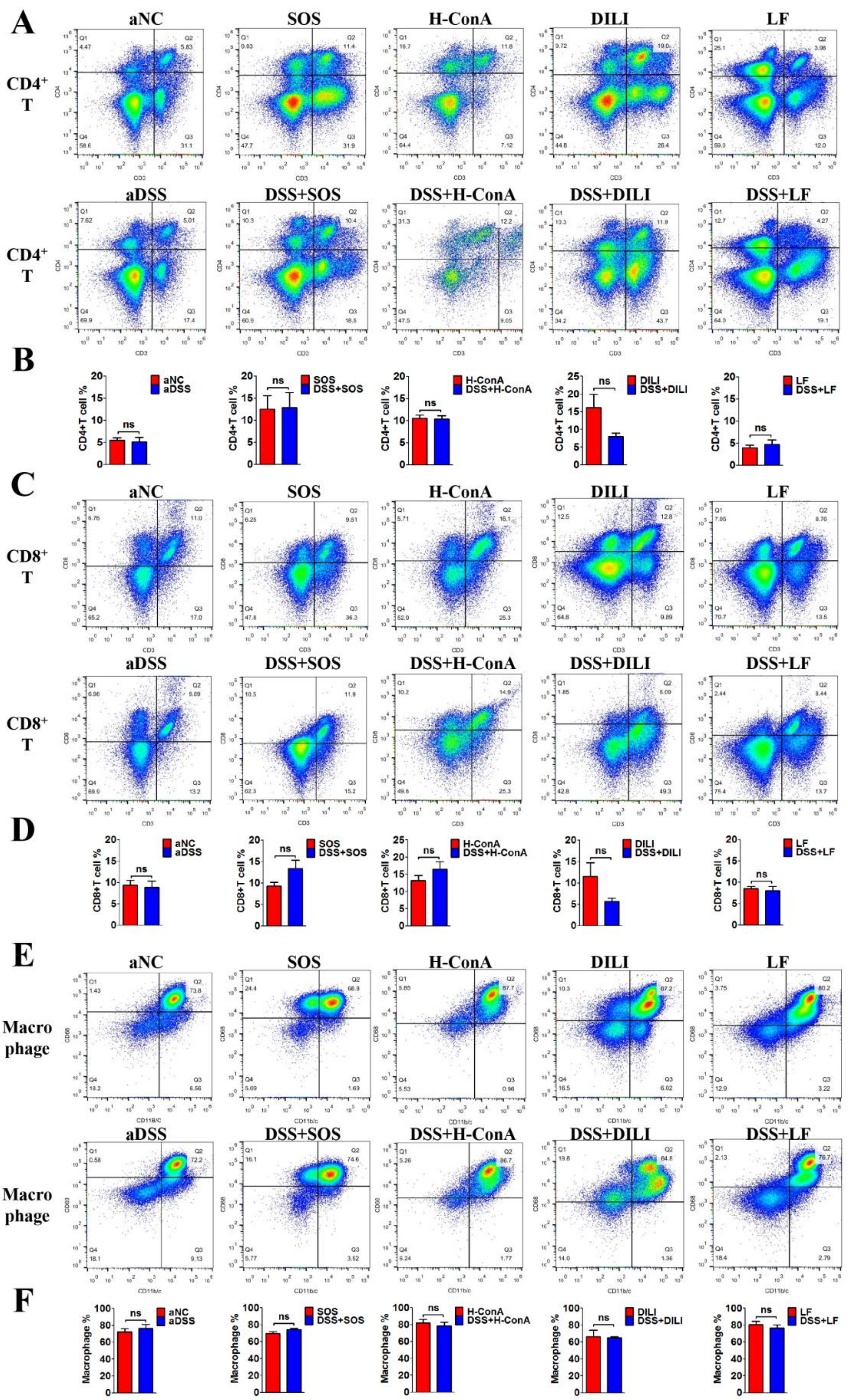

Fig. S9 Evaluation of the number of immunocytes in colitis-induced liver injury. The number of **(A** and **B)** $CD3^{+}CD4^{+}$T cells, **(C** and **D)** $CD3^{+}CD8^{+}$T cells and **(E** and

F) macrophages in colitis-induced liver injury were evaluated by flow cytometry. Abbreviation: NC, normal control; aDSS, acute dextran sulfate sodium-colitis; SOS, sinusoidal obstruction syndrome; H-ConA, high-dose ConA hepatitis; DILI, drug-induced liver injury; LF, liver fibrosis. *ns* means non-significant.

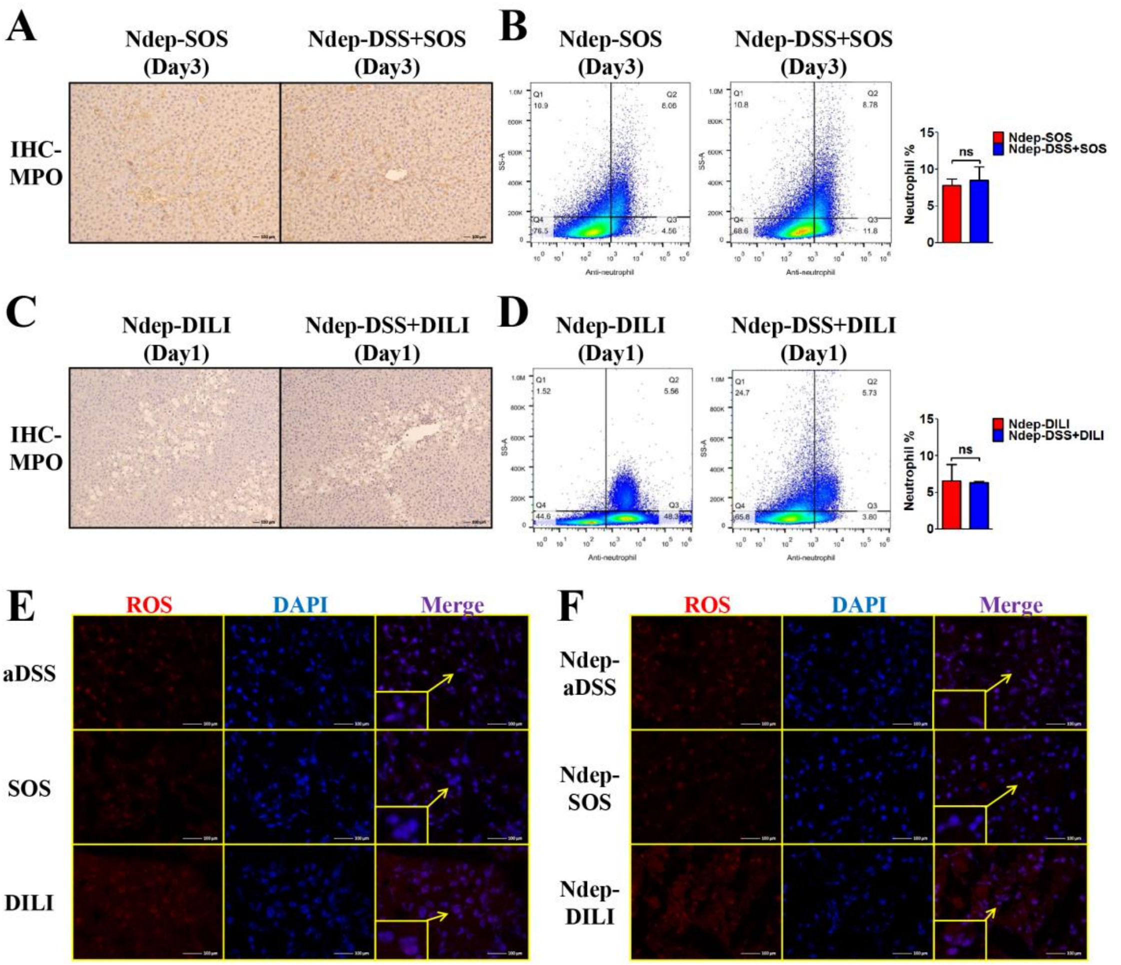

Fig. S10 The effect of neutrophils depletion and the role of neutrophils in colitis-induced liver injury.

After neutrophils deletion, the infiltration and number of hepatic neutrophils were evaluated by immunohistochemistry (IHC) for myeloperoxidase (MPO) and flow cytometry (Anti-neutrophil^{+}SSA^{high}) in **(A** and **B)** SOS model and **(C** and **D)** DILI model. **(E** and **F)** Immunofluorescence staining of reactive oxygen species (ROS) (red) also showed good correlation with the number of hepatic neutrophils. Abbreviation: Ndep, neutrophils depletion; SOS, sinusoidal obstruction syndrome; DSS, dextran sulfate sodium-colitis; DILI, drug-induced liver injury. ns means non-significant.

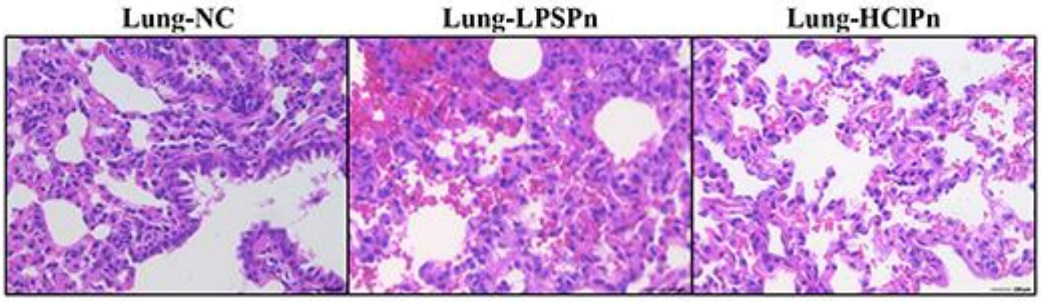

Fig. S11 H&E staining of lung.

H&E staining of the lungs in lipopolysaccharide-induced pneumonia (LPSPn) and hydrochloric acid-induced pneumonia (HClPn) showed alveolar hemorrhage, inflammation and destruction of alveolar structure as compared with normal control group (NC).

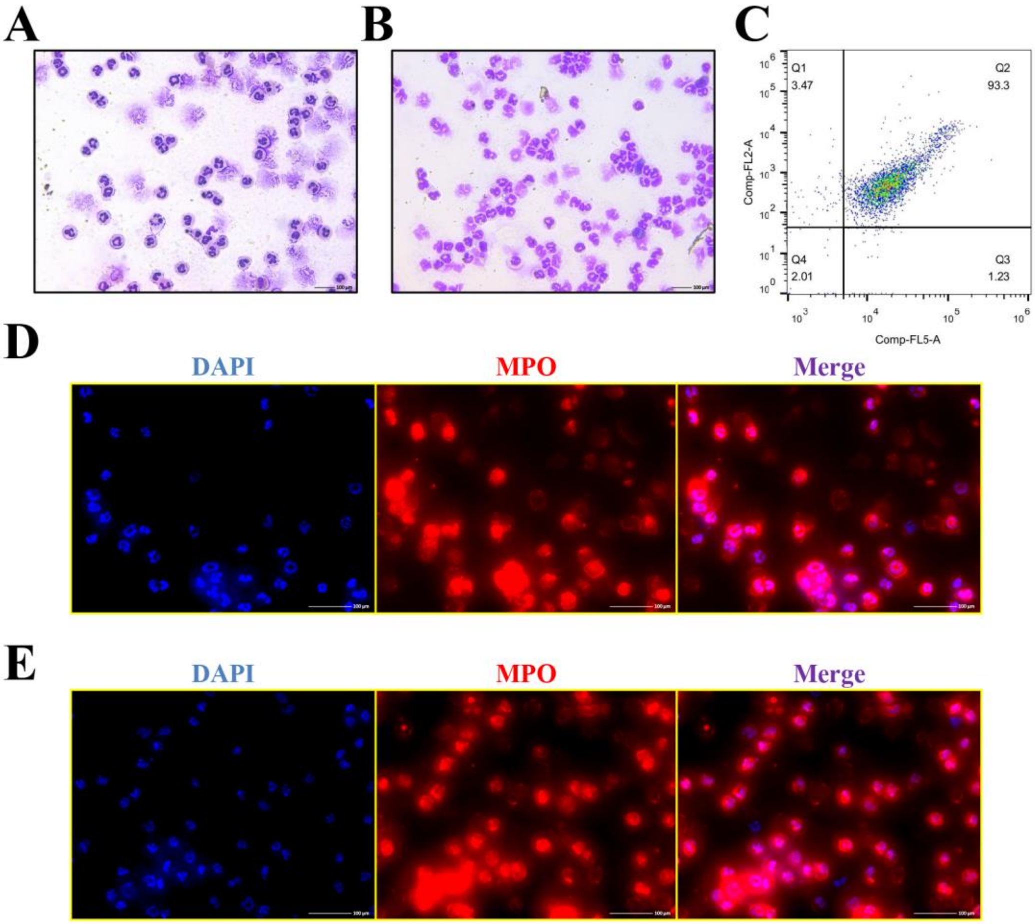

Fig. S12 Identification of isolated neutrophils and the influence of CXCL1 on neutrophil chemotaxis.

Giemsa staining was used to evaluate the morphology of isolated neutrophils from **(A)** peripheral blood and **(B)** the liver. **(C)** Flow cytometry (CD11b/c^{+}anti-neutrophil^{+}) was also used to test the purity of isolated peripheral blood neutrophils. Immunocytochemistry also show typical rod and lobulated shape of nucleus (as shown by 4',6-Diamidino-2-Phenylindole (DAPI)) as well as positive staining of myeloperoxidase (MPO) in neutrophils isolated from **(D)** the peripheral blood and **(E)** the liver.

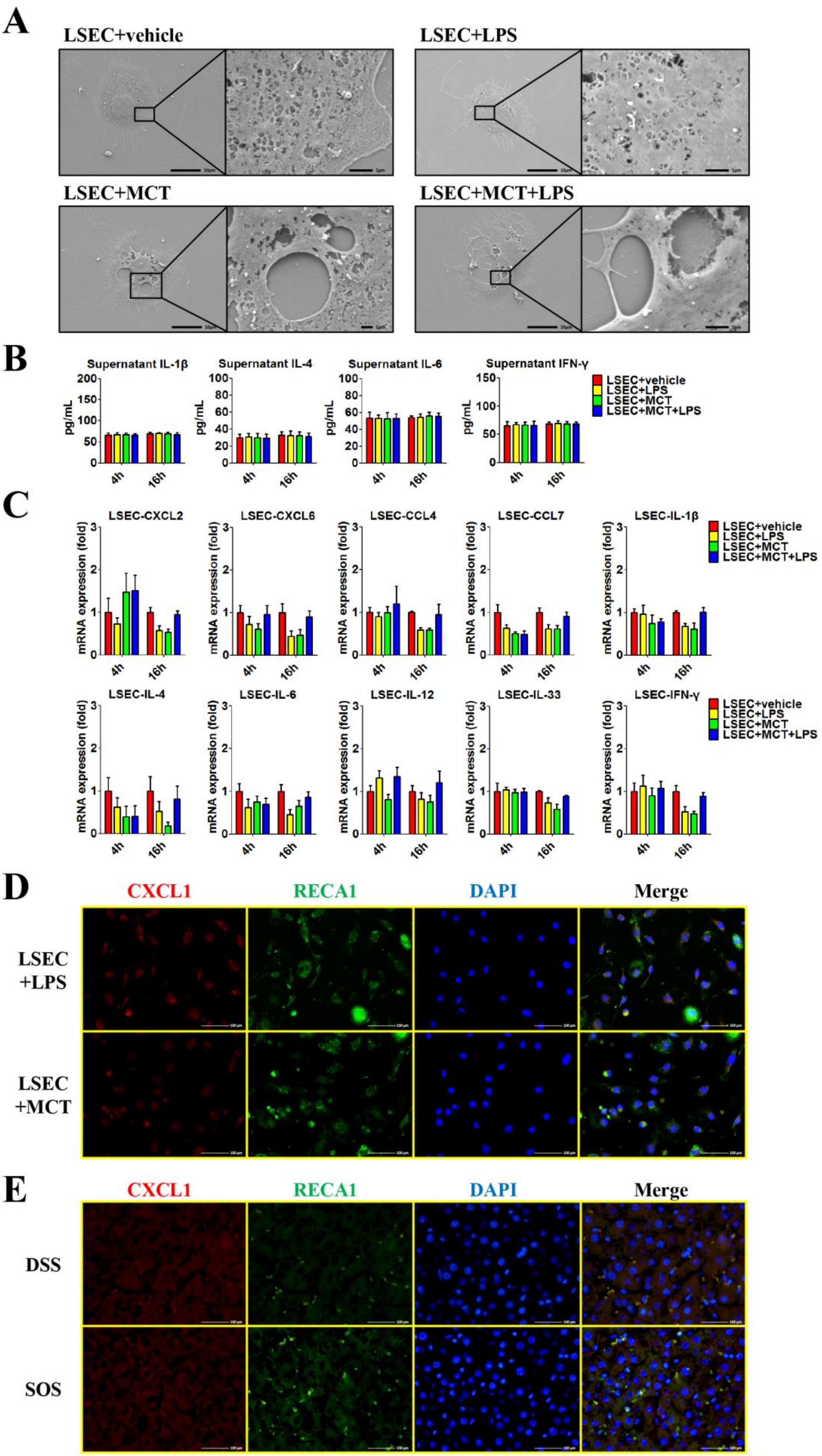
A
LSEC+vehicle
LSEC+LPS
LSEC+MCT
LSEC+MCT+LPS
B
Supernatant IL-1β
Supernatant IL-4
Supernatant IL-6
Supernatant IFN-γ
pg/mL
4h
16h
LSEC+vehicle
LSEC+LPS
LSEC+MCT
LSEC+MCT+LPS
C
LSEC-CXCL2
LSEC-CXCL6
LSEC-CCL4
LSEC-CCL7
LSEC-IL-1β
LSEC-IL-4
LSEC-IL-6
LSEC-IL-12
LSEC-IL-33
LSEC-IFN-γ
mRNA expression (fold)
D
CXCL1
RECA1
DAPI
Merge
LSEC +LPS
LSEC +MCT
E
DSS
SOS

325

Fig. S13 Features of damaged liver sinusoidal endothelial cells (LSECs) *ex vivo* and functional changes of LSECs.

(A) After LSECs were challenged by monocrotaline (MCT) and lipopolysaccharide (LPS), the morphological characteristics of LSECs were evaluated by scanning electron microscope (SEM). Normal LSECs showed a pattern mimicking rat liver sinusoidal endothelium, with intact lining and well-organized fenestrae of uniform size; while MCT induced obvious LSECs injury with distinct discontinuous cell surface and large gaps. LPS had no significant influence on the injury of LSECs. After LSECs were treated with different stimuli, the following parameters were examined: **(B)** the supernatant cytokines tested by ELISA and **(C)** mRNA levels of cytokines. **(D)** LSECs-derived CXCL1 was also examined by immunocytochemistry of CXCL1 (red) and RECA-1 (green, a marker of rat endothelial cells). **(E)** The co-localization of CXCL1 (red) and RECA-1 (green) was detected by immunohistochemistry in animal models.

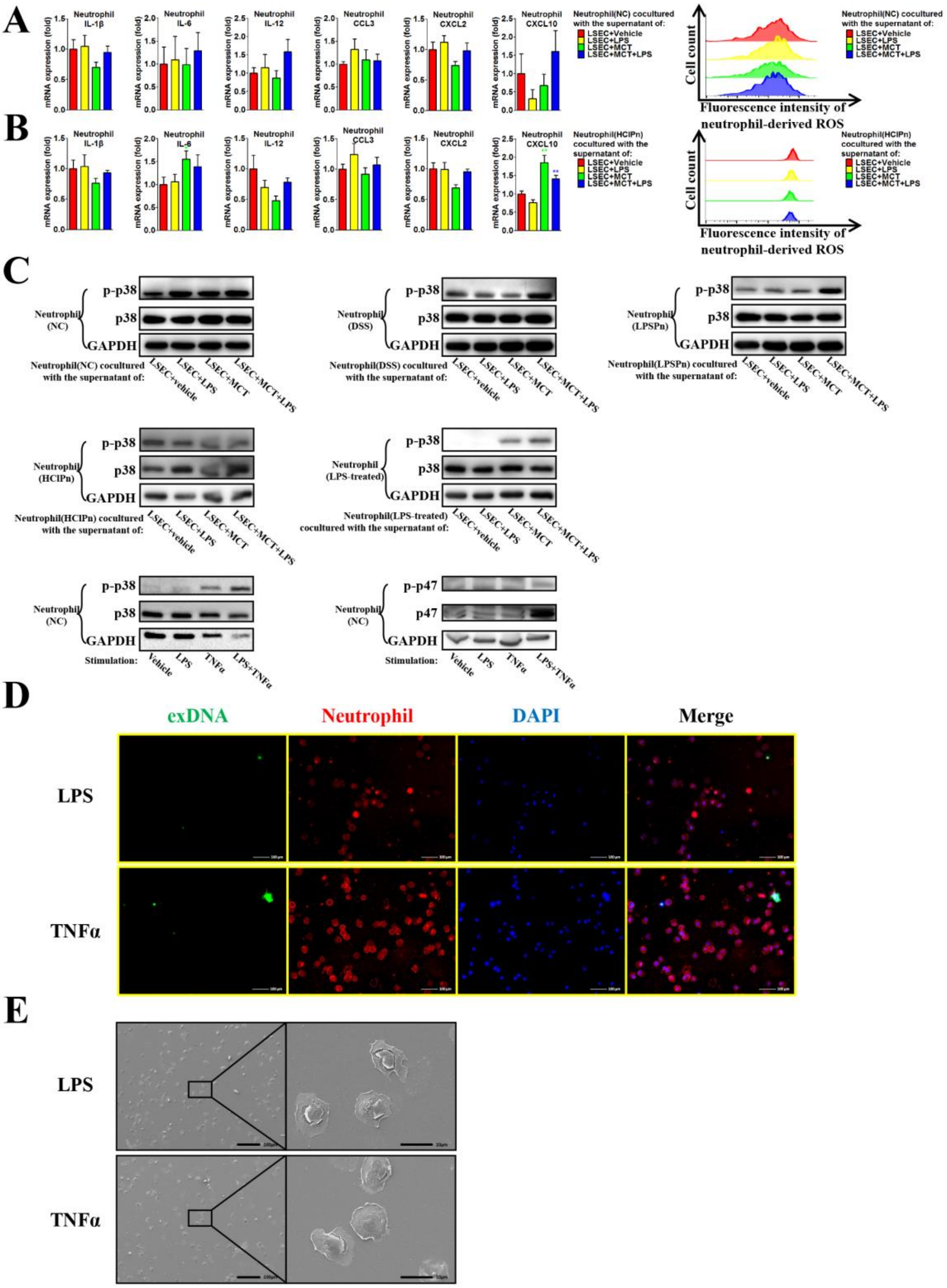

Fig. S14 Liver sinusoidal endothelial cells (LSECs) and lipopolysaccharide (LPS) contribute to neutrophil activation.

The activation status of neutrophils was evaluated via neutrophil-derived cytokines and neutrophil-derived reactive oxygen species (ROS) in the following experiments: **(A)** Neutrophils were isolated from NC rats and incubated with the supertants of LSECs under different treatments (vehicle, LPS, monocrotaline (MCT), MCT+LPS); **(B)** Neutrophils were isolated from HCl-induced pneumonia (LPSPn) rats and incubated with the supertants of LSECs. **(C)** Phosphorylation of p38 was tested in all

groups. Phosphorylation of p47 was also tested in neutrophils treated by LPS and TNF-α. **(D)** Neutrophil extracellular traps (NETs) were detected by immunocytochemistry of neutrophil-derived extracellular DNA (exDNA, green). **(E)** Scanning electron microscopy was used to evaluate the morphology of neutrophils.

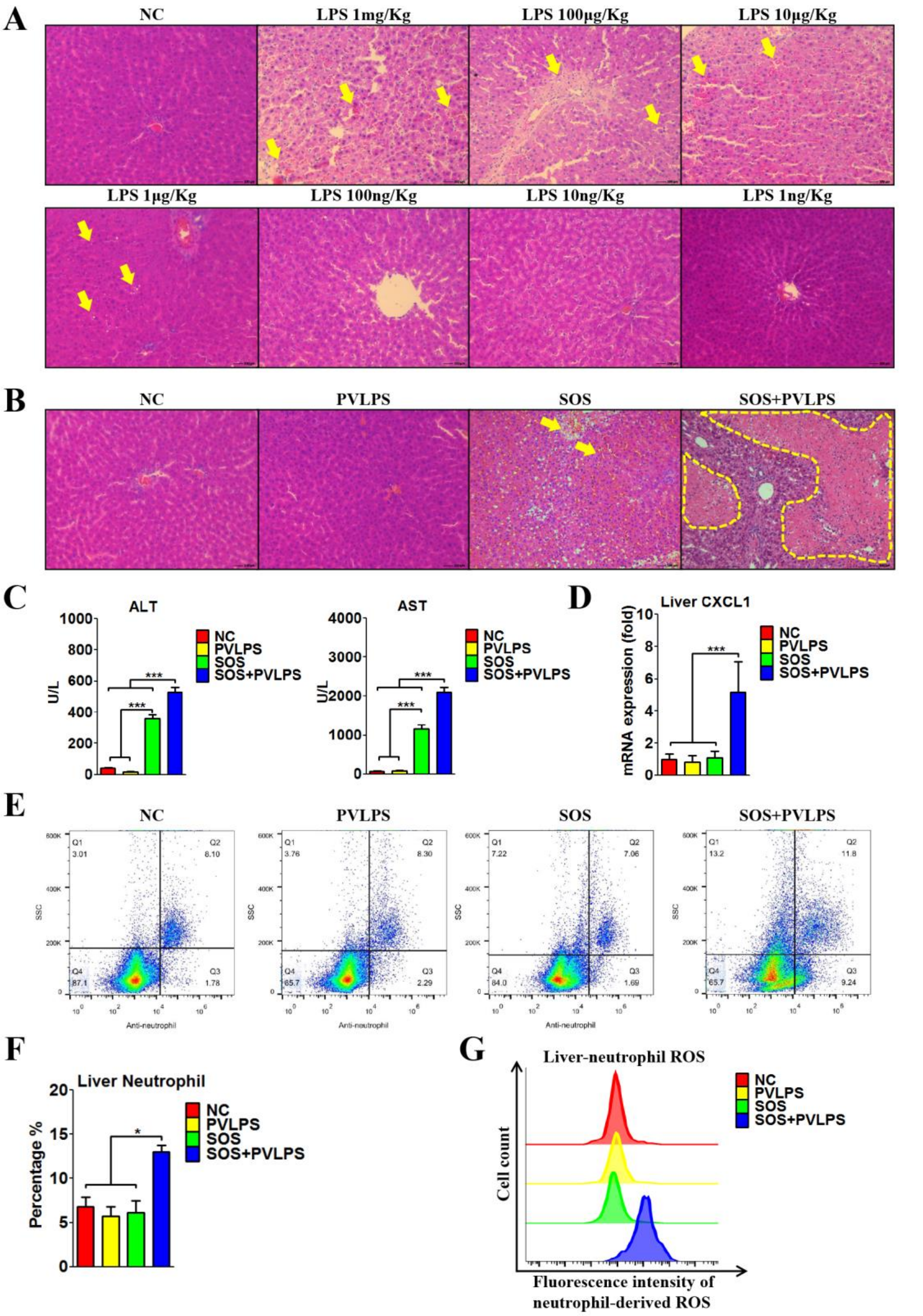

Fig. S15 The effect of LPS injection in the portal vein.

(A) To induce a model (PVLPS) with increased LPS level in the portal vein, a dose

gradient of LPS was injected in the portal vein, and the pathological changes of liver were evaluated after 24h. The effect of PVLPS (100ng/Kg) on liver injury was evaluated in sinusoidal obstruction syndrome (SOS) model via **(B)** H&E staining of the liver, **(C)** alanine aminotransferase (ALT) and aspartate aminotransferase (AST), **(D)** liver CXCL1 mRNA level, **(E** and **F)** flow cytometry of hepatic neutrophils, and **(G)** hepatic neutrophils-derived reactive oxygen species (ROS). *, $P<0.05$; ***, $P<0.001$.
